# Histone methyltransferase SET-18/SMYD2-mediated activation of NADase TIR-1d/SARM1 increases mtROS to promote aging

**DOI:** 10.1016/j.isci.2026.114649

**Published:** 2026-01-07

**Authors:** Dongxue Xue, Xin Su, Aaron Pambu Lelo, Yongjun Zhang, Xueqing Ba, Cheng-gang Zou, Aohe Ma, Yao Liu, Xiaoxue Li

**Affiliations:** 1 Key Laboratory of Molecular Epigenetics of the Ministry of Education, School of Life Science, Northeast Normal University, Changchun, Jilin 130024, China; 2 State key Laboratory for Conservation and Utilization of Bio-Resources in Yunnan, School of Life Sciences, Yunnan University, Kunming, Yunnan 650091, China; 3 Department of Life Science, Faculty of Science and Technologies, University of Kinshasa, Kinshasa, Democratic Republic of the Congo

**Keywords:** Biochemistry, Molecular biology, Cell biology

## Abstract

Histone lysine methylation regulates the expressions of mitochondrial function-related genes, which presents a “nucleus-to-mitochondria” signal communication, playing a key role in aging control. However, the underlying mechanisms remain elusive due to the complexity of histone lysine methylation in transcription modulation. In this study, using *C. elegans* and mouse C2C12 cell-differentiated myotubes as research models, we found that histone H3K36me2 methyltransferase SET-18/SMYD2 were responsible for the increase of mitochondrial reactive oxygen species (mtROS) accumulation during aging. Mechanistically, SET-18/SMYD2-mediated H3K36me2 modification upregulated the expression of NADase *tir-1 isoform d* (*tir-1d*)/*sarm1* to decrease NAD^+^ level. Consequently, mtROS level was elevated, which resulted in shortened worm lifespan as well as accelerated mouse myotubes atrophy (a hallmark of muscle aging). These findings proposed that mtROS generation is actively regulated other than passively accumulated in aging process, and revealed a “H3K36me2-NADase-mtROS” signaling axis of “nucleus-to-mitochondria” communication to modulate aging, which is conserved from *C. elegans* to mammals.

## Highlights

• SET-18/SMYD2 increases mtROS level to promote aging of *C. elegans* and mouse muscle
• SET-18/SMYD2 targets NADase TIR-1d/SARM1 to enhance mtROS accumulation to accelerate aging
• SET-18/SMYD2 activates NADase TIR-1d/SARM1 expression through histone H3K36me2 modification

## Graphical abstract

**Figure:**
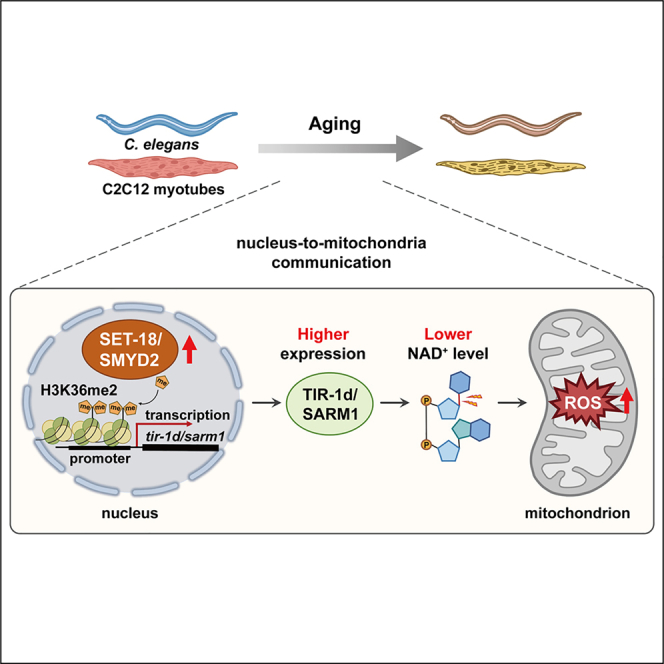

## Introduction

Aging, characterized by the functional decline of tissues and organs, usually raises the susceptibility to a range of human diseases and disorders, including neurodegenerative diseases, diabetes, cardiovascular disorders and cancer^1^^,^^2^ Epigenetic alterations, including DNA methylation, histone post-translational modifications, and chromatin organization and remodeling, contribute to reprogramming the transcriptional profiling in older tissues of the organisms, which has been identified as one of the major hallmarks of aging.^3^^,^^4^ Histone methylation may occur at the side chains of different amino acid residues such as lysine (Lys) or arginine,^5^ with an exquisite site selectivity for Lys methylation at specific positions in the N-termini of histones.^6^ In contrast to histone acetylation or lactylation, lysine methylation of histone is responsible for both repression and activation of transcription, depending on the particular histone being modified, the residue, and the number of methyl groups.^7^ Therefore, although accumulating evidence from yeast, *C. elegans*, to mammals has shown that histone lysine methylation is closely linked to aging,^8^ the diversity and complexity of this modification in gene regulation prevent a thorough understanding the mechanism of its function in aging control.

A causative link between mitochondrial dysfunction and aging has been established for decades.^9^ Particularly, mitochondrial reactive oxygen species (mtROS) is taken as one of mitochondrial stress signalings that play key roles in longevity modulation.^10^^,^^11^ Accumulating evidence is illustrating the roles of histone methylation in the expressional regulation of mitochondrial function-related genes.^12^^,^^13^ For example, the genes that stimulate mitochondrial unfolded protein response (UPR^mt^, the other kind of mitochondrial stress signaling), such as the chaperone *hsp-6* and transcriptional regulator *dve-1*, are discovered to be the novel targets of histone lysine methyltransferases and demethylases during longevity control.^12^^,^^13^ However, the implication of histone lysine methyltransferases and demethylases in regulation of the genes that modulate mtROS generation to affect aging remains poorly understood.

SMYD2, a member of SMYD (SET and MYND domain-containing proteins) family in mammals, is first identified as a histone H3K36me2 methyltransferase.^14^ However, a number of evidence has shown that SMYD2 play roles in development and cancer primarily by catalyzing the methylation of non-histone proteins.^15^ For example, SMYD2-mediated methylation of Hsp90 influences cardiac contraction and myofilament organization.^16^^,^^17^ SMYD2 accelerates cell proliferation and migration by methylation of P53, RB, PTEN, and ERα in several types of cancer, including breast cancer, esophageal squamous cell carcinoma, and blander cancer.^18^^,^^19^^,^^20^^,^^21^ Therefore, the functions of SMYD2-catalyzed histone H3K36me2 modification in reprogramming the transcriptional profiling and the consequent outcomes in cellular and biological process remain to be further revealed.

Previously, we have reported that SET-18, the *C. elegans* homologous of SMYD2, contributes to shortening lifespan by repressing the expression of *daf-16a*, the key transcriptional factor to activate a series of anti-oxidative genes,^22^ suggesting a contribution of SET-18/SMYD2 targets in accumulation of cellular ROS. In this study, *C. elegans* NADase TIR-1d, the homologous of mammal SARM1, is identified as a key target of SET-18/SMYD2. Nicotinamide adenine dinucleotide (NAD^+^), a crucial metabolite generated in mitochondria, is a well-known longevity-promoting molecule.^23^^,^^24^ It has been reported that decline of NAD^+^ level is able to induce the increase of NADH/NAD^+^ ratio,^25^^,^^26^ which leading to improving the reduction of flavin mononucleotide (FMN) on complex I, thereby raising mtROS production.^27^ Therefore, we propose that SET-18/SMYD2 may modulate the expressions of TIR-1d/SARM1 by histone H3K36me2 modification, thereby enhancing mtROS generation, and consequently leading to acceleration of aging. Our present work unveiled that “H3K36me2-NADase-mtROS” represents a conservative signaling axis of “nucleus-to-mitochondria” communication to control aging from worms to mammals.

## Results

### SET-18 increases mtROS accumulation to reduce viability of *C. elegans*

**Figure 1:**
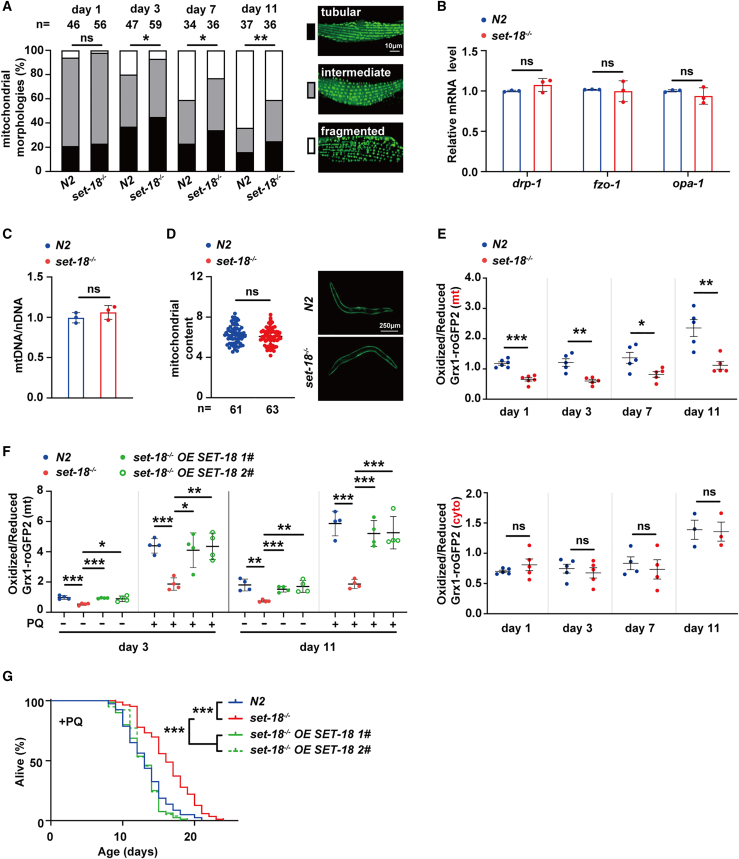
SET-18 increases mtROS accumulation to reduce viability of *C. elegans* (A) Loss of *set-18* reduced mitochondrial fragmentation during worm aging. The *P*_*myo-3*_::mito::GFP reporters were used for detecting the mitochondrial network in the muscle cells of N2 and *set-18* mutants from young (day 1 and day 3) to old (day 7 and day 11) ages by confocal microscopy. According to previous publication,^28^ The mitochondrial morphology of the muscle cells were classified into three types, including long tubular (Black), intermediate (Gray) and fragmented (White) (on the left), with the representative images shown on the right. n, the number of muscle cells used for analyzing mitochondrial morphology of each strain. ns, no significant difference. Day 1: p (*set-18*^−/−^ vs. N2) = 0.3468, day 3: ∗p (*set-18*^−/−^ vs. N2) = 0.0258, day 7: ∗p (*set-18*^−/−^ vs. N2) = 0.0202, day 11: ∗∗p (*set-18*^−/−^ vs. N2) = 0.0049; *chi*-square. (B) The mRNA expression levels of mitochondrial fusion and fission genes were not altered by mutation of *set-18*. The mRNA levels of mitochondrial fusion genes (*fzo-1*, *opa-1)* and fission gene (*drp-1*) of N2 and *set-18* mutants at day 3 were measured by RT-qPCR and normalized to that of N2. *act-1* was used as an internal reference. Error bars represent standard error of the mean (SEM). *n* = 3 biological replicates. *drp-1*: p (*set-18*^−/−^ vs. N2) = 0.1883, *fzo-1*: p (*set-18*^−/−^ vs. N2) = 0.7904, *drp-1*: p (*set-18*^−/−^ vs. N2) = 0.3389; Student’s *t* test. (C–D) The mitochondrial abundance was not changed by deletion of *set-18*. (C) The ratio of copy number of mitochondrial DNA (mtRNA) to nuclear DNA (nDNA) was detected RT-qPCR. *n* = 3 biological replicates. p (*set-18*^−/−^ vs. N2) = 0.3393; Student’s *t* test. (D) The mitochondrial content was quantified by analyzing fluorescence intensity of *P*_*myo-3*_::mito::GFP reporters in the mitochondria of worms. The GFP expression in N2 and *set-18* mutants were detected by fluorescence microscopy with the identical exposure settings, with the representative images shown in this figure. Error bars represent SEM. n, the number of worms used for quantitative analysis of fluorescence intensity. p (*set-18*^−/−^ vs. N2) = 0.2567; Student’s *t test*. (E) Loss of *set-18* decreased mtROS accumulation during worm aging. The mitochondrial (mt) and cytoplasmic (cyto) ROS levels of N2 and *set-18* mutants with Grx1-roGFP2 expressions at young (day 1 and day 3) and old (day 7 and day 11) ages were measured by fluorescence microplate reader, respectively. The redox ratio was defined as the fluorescence intensity of oxidized Grx1-roGFP2 (excited at 405 nm) divided by that of reduced Grx1-roGFP2 (excited at 488 nm) with corrections for worms’ intestinal autofluorescence. Error bars represent SEM. n = 3–5 biological replicates. Day 1 mtROS: ∗∗∗p (*set-18*^−/−^ vs. N2) < 0.0001, day 3 mtRO**S**: ∗∗p (*set-18*^−/−^ vs. N2) = 0.0019, day 7 mtROS: ∗p (*set-18*^−/−^ vs. N2) = 0.0276, day 11 mtROS:∗∗p (*set-18*^−/−^ vs. N2) = 0.0037, day 1 cytoROS: p (*set-18*^−/−^ vs. N2) = 0.3060, day 3 cytoROS: ∗∗p (*set-18*^−/−^ vs. N2) = 0.5469, day 7 cytoROS: ∗p (*set-18*^−/−^ vs. N2) = 0.6156, day 11 cytoROS:∗∗p (*set-18*^−/−^ vs. N2) = 0.8968; Student’s *t* test. (F) Rescue of *set-18* attenuated the decline of mtROS level in *set-18* mutants with/without paraquate treatment. “*set-18*^−/−^*OE SET-18*” *1#* and *2#* were two independent extrachromosomal arrays obtained by co-injecting plasmids *P*_*set-18*_::SET-18::mCherry with *rol-6* marker (pRF4) into *set-18* mutants. The mtROS levels of worms at young (day 3) and old (day 11) ages with/without 2 mM paraquate (PQ) treatment were assessed using the fluorescence intensity ratio of oxidized Grx1-roGFP2 to reduced ones in mitochondria. Error bars represent SEM. *n* = 4 biological replicates. Day 3: ∗∗∗p (*set-18*^−/−^ vs. N2) = 0.0008, ∗∗∗p (*set-18*^−/−^*OE SET-18 1#* vs. *set-18*^−/−^) < 0.0001, ∗p (*set-18*^−/−^*OE SET-18 2#* vs. *set-18*^−/−^) = 0.0117; day 3+PQ: ∗∗∗p (*set-18*^−/−^ vs. N2) = 0.0002, ∗p (*set-18*^−/−^*OE SET-18 1#* vs. *set-18*^−/−^) = 0.0102, ∗∗p (*set-18*^−/−^*OE SET-18 2#* vs. *set-18*^−/−^) = 0.0022; day 11: ∗∗p (*set-18*^−/−^ vs. N2) = 0.0018, ∗∗∗p (*set-18*^−/−^*OE SET-18 1#* vs. *set-18*^−/−^) = 0.0003, ∗∗p (*set-18*^−/−^*OE SET-18 2#* vs. *set-18*^−/−^) = 0.0032; day 11+PQ: ∗∗∗p (*set-18*^−/−^ vs. N2) < 0.0001, ∗∗∗p (*set-18*^−/−^*OE SET-18 1#* vs. *set-18*^−/−^) = 0.0003, ∗∗∗p (*set-18*^−/−^*OE SET-18 2#* vs. *set-18*^−/−^) = 0.0009; one-way ANOVA. (G) Overexpression of *set-18* abolished the increased viability of *set-18* mutants with paraquate treatment. All of worms were treated with 2 mM paraquate (PQ). The survivals of worms were scored based on three independent experiments, with representative examples shown in this figure. ∗∗∗p (*set-18*^−/−^ vs. N2) < 0.0001, ∗∗∗p (*set-18*^−/−^*OE SET-18 1#* vs. *set-18*^−/−^) < 0.0001, ∗∗∗p (*set-18*^−/−^*OE SET-18 2#* vs. *set-18*^−/−^) < 0.0001; *log rank* test. All data and statistical analyses are provided in Table S1.

To address whether mitochondrial dysfunction and mtROS accumulation account for the aging-promoting effects of SET-18, firstly, we detected mitochondrial morphology in wild-type (N2) and *set-18* mutant worms from young age (day 1 and day 3) to old age (day 7 and day 11) by utilizing *P*_*myo-3*_::mito::GFP reporter that expresses the mitochondria-targeted GFP in muscles. Results showed that in N2 worms, compared to day 1, the percentages of fragmented mitochondria increased at day 3 and this upregulation gradually enhanced from day 7 to day 11 (Figure 1A). And at day 3, day 7, and day 11, the fragmentation of mitochondria were all attenuated by deletion of *set-18* (Figure 1A). However, at day 3, the mRNA levels of mitochondrial fusion and fission genes (*fzo-1*, *opa-1*, and *drp-1*) were not altered in *set-18* mutants (Figure 1B); and loss of *set-18* also did not change the copy number of mtDNA (quantified by the ratio of mitochondrial DNA to nuclear DNA) (Figure 1C), as well as the mitochondrial content (measured by the mitochondria-targeted GFP fluorescence intensities) (Figure 1D). Therefore, we speculated that SET-18 might modulate fragmentation of mitochondria by changing mitochondrial stress signaling, such as mtROS and/or UPR^mt^.

The mitochondrial and cytoplasmic ROS level in *C. elegans* were quantified using two transgenic strains *Pmyo3::mt::Grx1-roGFP2* and *Pmyo3::cyto::Grx1-roGFP2* that express the redox-sensitive fusion protein “Grx1-roGFP2” specifically in the mitochondria and cytoplasm of muscular cells, respectively, as described previously.^29^^,^^30^^,^^31^ ROS measurements were performed via fluorescence microplate reader due to its advantage to exclude the intestinal autofluorescence of worms, with the redox ratio defined as the fluorescence intensity of oxidized Grx1-roGFP2 (excited at 405 nm) divided by that of reduced Grx1-roGFP2 (excited at 488 nm). The redox ratio analysis showed that throughout the whole worm life (from day 1 to day 11), the ROS level in mitochondria, but not in cytoplasm, was decreased by deletion of *set-18*, although both of mitochondrial and cytoplasmic ROS levels in N2 worms at older stages (day 7 and day 11) were higher than those at the young ages (day 1 and day 3) (Figure 1E). However, compared with N2 worms, the expression level of *hsp-6p*::GFP (the UPR^mt^ reporter) was not changed in *set-18* mutants, no matter whether UPR^mt^ was activated by *cco-1* RNAi treatment or not (Figure S1). These suggested that SET-18 is particularly responsible for increasing mitochondrial ROS (mtROS) accumulation.

To further verify the effect of SET-18 on increasing mtROS production, 2 mM paraquate (PQ) was used to specifically raise mtROS level in worms (Figure S2) as described previously.^32^ We found that loss of *set-18* attenuated the PQ-induced upregulation of mtROS in both young (day 3) and old (day 11) worms (Figure 1F). Survival assays showed that the *set-18* mutants lived longer than N2 worms while they were treated with 2 mM PQ (Figures 1G, Table S1); and the mean survival (12.898 ± 0.179 days) of N2 worms with 2 mM PQ treatment was consistent to that in previous publication.^32^ Moreover, overexpression of SET-18 driven by its own promoter not only attenuated the decline of mtROS level in *set-18* mutants with PQ treatment (Figure 1F), but also alleviated the up-regulation of these worms’ viability (Figure 1G). These findings together implied that SET-18 promotes *C. elegans* aging by increase of mtROS accumulation.

### SET-18-mediated alteration in NAD^+^ metabolism contributes to mtROS accumulation to promote aging

**Figure 2:**
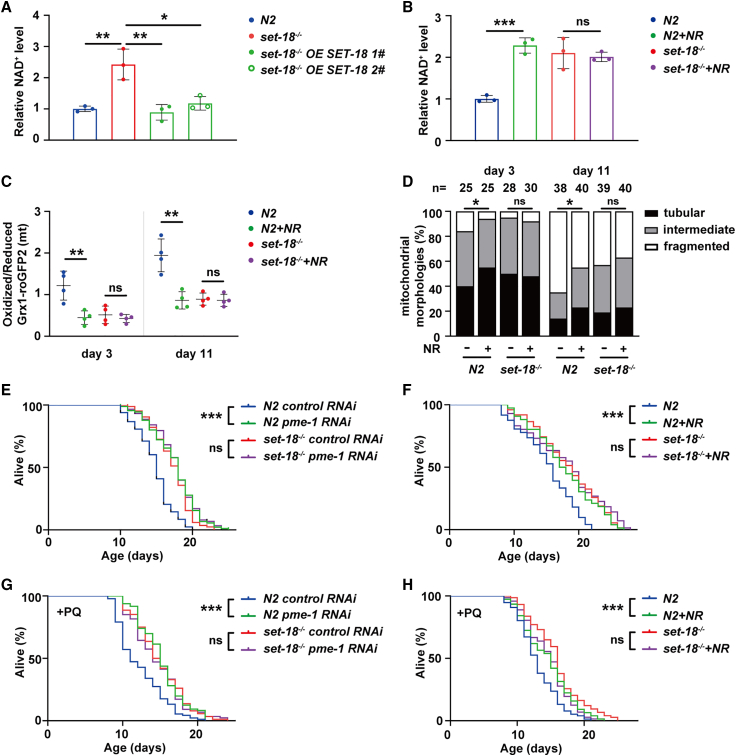
SET-18-mediated alteration in NAD^+^ metabolism contributes to mtROS accumulation to promote worm aging (A) SET-18 was responsible for decreasing NAD^+^ level in worms. The strains “*set-18*^−/−^*OE SET-18*” *1#* and *2#* were identical to the ones used in Figure 1G. The NAD^+^ level of worms were detected by microplate reader and normalized to that of N2. Error bars represent SEM. *n* = 3 biological replicates. ∗∗p (*set-18*^−/−^ vs. N2) = 0.0079, ∗∗p (*set-18*^−/−^*OE SET-18 1#* vs. *set-18*^−/−^) = 0.0086, ∗p (*set-18*^−/−^*OE SET-18 2#* vs. *set-18*^−/−^) = 0.0161; one-way ANOVA. (B) The NR-induced up-regulation of NAD^+^ levels was not observed in *set-18* mutants. N2 and *set-18* mutant worms (day 1) were treated with 500 μM NR, a precursor of NAD^+^ (re-)synthesis. Error bars represent SEM. *n* = 3 biological replicates. ∗∗∗p (N2+NR vs. N2) = 0.0004, p (*set-18*^−/−^ + NR vs. *set-18*^−/−^) = 0.7006; Student’s *t* test. (C and D) The functions of NR in reducing mtROS level and decreasing mitochondrial fragmentation during worm aging were both attenuated by loss of *set-18*. (C) The analysis of mtROS level. The mtROS level of N2 and *set-18* mutant worms (day 3 and day 11) with/without 500 μM NR treatment were measured by analyzing the ratio of oxidized Grx1-roGFP2 to reduced ones in mitochondria. *n* = 4 biological replicates. Day 3: ∗∗p (N2+NR vs. N2) = 0.0070, ns = no significant difference, p (*set-18*^−/−^ + NR vs. *set-18*^−/−^) = 0.4792; day 11: ∗∗p (N2+NR vs. N2) = 0.0028, p (*set-18*^−/−^ + NR vs. *set-18*^−/−^) = 0.7862; Student’s *t* test. (D) The analysis of mitochondrial morphology. N2 and *set-18* mutant worms (day 3 and day 11) expressing *P*_*myo-3*_::mito::GFP reporters were treated with/without 500 μM NR, respectively. The mitochondrial morphology of muscle cells in worms were detected and classified according to the legend of Figure 1B n, the number of muscle cells used for analysis of mitochondrial morphology. Error bars represent SEM. Day 3: ∗p (N2+NR vs. N2) = 0.0271, p (*set-18*^−/−^ + NR vs. *set-18*^−/−^) = 0.7265; day 11: ∗p (N2+NR vs. N2) = 0.0173, p (*set-18*^−/−^ + NR vs. *set-18*) = 0.6433; Student’s *t* test. (E–H) Mutation of *set-18* alleviated the effects of *pme-1* RNAi and NR treatment in enhancing mtROS-defense ability and extending lifespan. N2 and *set-18* mutant worms were treated with *pme-1* RNAi (E and G) and 500 μM NR (F and H), respectively. (E and F) The lifespan assays. (G–H) The mtROS-defense assays. The worms with 2 mM PQ treatment were used for survival analysis. All of assays were scored based on three independent experiments, with representative examples shown in this figure. Lifespan assays: ∗∗∗p (*N2 pme-1 RNAi* vs. *N2 control RNAi*) < 0.0001, p (*set-18*^−/−^*pme-1 RNAi* vs. *set-18*^−/−^*control RNAi*) = 0.6150; ∗∗∗p (N2+NR vs. N2) < 0.001, p (*set-18*^−/−^ + NR vs. *set-18*^−/−^) = 0.1138; PQ survival assays: ∗∗∗p (*N2 pme-1 RNAi* vs. *N2 control RNAi*) < 0.0001, p (*set-18*^−/−^*pme-1 RNAi* vs. *set-18*^−/−^*control RNAi*) = 0.5447; ∗∗∗p (N2+NR vs. N2) < 0.0001, p (*set-18*^−/−^ + NR vs. *set-18*^−/−^) = 0.3436; *log rank* test. All data and statistical analyses are provided in Table S2.

Mitochondrial reactive oxygen species (mtROS) are primarily generated through electron leakage from the mitochondrial electron transport chain (ETC), particularly during the reduction of complex I’s flavin mononucleotide (FMN) via electron transfer from NADH.^33^^,^^34^ Notably, lowering the NADH/NAD^+^ ratio by elevating NAD^+^ level can suppress mtROS generation.^27^^,^^35^ Therefore, the NAD^+^ levels in N2 worms and *set-18* mutants were measured and the results showed that loss of *set-18* raised NAD^+^ level, and this increase was attenuated by rescue of SET-18 expression (Figure 2A). As numerous evidence has shown that NAD^+^ level significantly declines during worm aging,^36^^,^^37^^,^^38^ we verified this results (Figure S3) and then fed the worms with nicotinamide riboside, a precursor of NAD^+^ (re-)synthesis to elevate NAD^+^ level. 500 μM NR was used because it functioned effectively, but not yet reached a saturation plateau (Figure S4). And we found that the NR-induced upregulation of NAD^+^ levels was not observed in *set-18* mutants (Figure 2B).

Next, the mtROS level was measured by utilizing the worms expressing mitochondrial Grx1-roGFP2 reporter as described in Materials and Methods. Results showed that in both young (day 3) and old (day 11) worms, supplementation of NR downregulated the mtROS level in N2 worms, but not in *set-18* mutants (Figure 2C). And the change of mitochondrial fragmentation in these worms showed similar to the tendency of mtROS level (Figure 2D). Additionally, we found that the mRNA expression levels of the ETC complex genes were not influenced by loss of *set-18* (Figure S5), implying that SET-18 might have no effect on the abundance of ETC complex proteins. The combined data suggested that SET-18 modulates NAD^+^ levels, which contributes to ROS production.

Moreover, we treated the worms with NR and *pme-1* RNAi, respectively, followed by lifespan and mtROS-defense assays. *Pme-1* is a NAD^+^-consuming gene encoding the ortholog of mammalian poly (ADP-ribose) polymerase 1 (PARP1) in *C. elegans*, and knockdown or deletion of *pme-1/parp1* are reported to conservatively increase NAD^+^ level in worms and mammals.^39^^,^^40^ Our results showed that RNAi of *pme-1* (Figure S6) and supplementation of NR both extended the lifespan of N2 worms (Figures 2E and 2F, Table S2) and enhanced their ability to defense against PQ-induced mtROS (Figures 2G and 2H, Table S2); and these effects of *pme-1* RNAi and NR treatment were alleviated by loss of *set-18* (Figures 2E–2H, Table S2). Collectively, these findings together implied that SET-18-mediated alteration in NAD^+^ metabolism contributes to mtROS accumulation to promote worm aging.

### SET-18 targets NADase TIR-1 to accelerate mtROS accumulation-mediated aging

**Figure 3:**
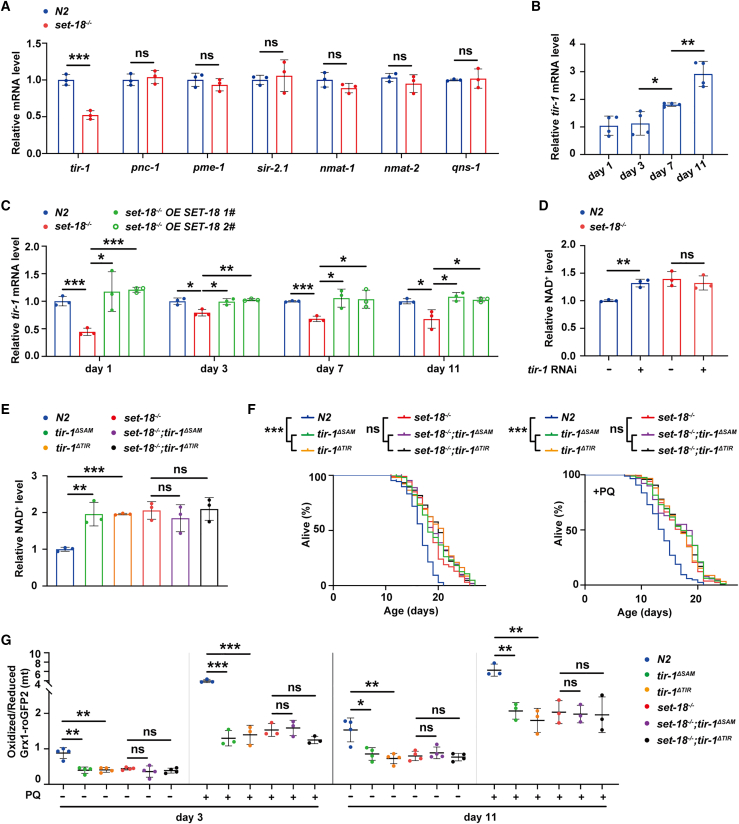
SET-18 enhances mtROS accumulation to accelerate aging by targeting a NADase TIR-1 (A) Loss of *set-18* decreased the mRNA level of *tir-1*. The mRNA levels of the key NAD^+^ consumption and biosynthesis genes, including *tir-1*, *pnc-1*, *pme-1*, *sir-2.1*, *nmat-1*, *nmat-2*, and *qns-1*, were measured by RT-qPCR and normalized to those of N2. *act-1* was used as an internal reference. Error bars represent the SEM. *n* = 3 biological replicates. *tir-1*: ∗∗∗p (*set-18*^−/−^ vs. N2) = 0.0009, *pnc-1*: p (*set-18*^−/−^ vs. N2) = 0.6123, *pme-1*: p (*set-18*^−/−^ vs. N2) = 0.3891, *sir-2.1*: p (*set-18*^−/−^ vs. N2) = 0.6842, *nmat-1*: p (*set-18*^−/−^ vs. N2) = 0.1699, *nmat-2*: p (*set-18*^−/−^ vs. N2) = 0.3333, *qns-1*: p (*set-18*^−/−^ vs. N2) = 0.8132; Student’s *t* test. (B) The *tir-1* mRNA level gradually increased from young (day 1 and day 3) to old age (day 7 and day 11) in N2 worms. The *tir-1* mRNA level in N2 at day 1, day 3, day 7, and day 11 were measured by RT-qPCR and normalized by that level at day 1. *act-1* was used as an internal control. Error bars represent SEM. *n* = 3 biological replicates. ∗p (day 7 vs. day 3) = 0.0196, ∗∗p (day 11 vs. day 7) = 0.0029; one-way ANOVA. (C) SET-18 was responsible for up-regulation of *tir-1* mRNA expression level through the whole worm life. The strains “*set-18*^−/−^*OE SET-18*” *1#* and *2#* were identical to the ones used in Figure 1G. The mRNA levels of *tir-1* in worms at day 1, day 3, day 7 and day 11 were measured by RT-qPCR and normalized to that of N2 at each age, respectively. *act-1* was used as an internal reference. Error bars represent SEM. *n* = 3 biological replicates. Day 1: ∗∗∗p (*set-18*^−/−^ vs. N2) = 0.0009, ∗p (*set-18*^−/−^*OE SET-18 1#* vs. *set-18*^−/−^) = 0.0261, ∗∗∗p (*set-18*^−/−^*OE SET-18 2#* vs. *set-18*^−/−^) < 0.0001; day 3: ∗p (*set-18*^−/−^ vs. N2) = 0.0120, ∗p (*set-18*^−/−^*OE SET-18 1#* vs. *set-18*^−/−^) = 0.0124, ∗∗p (*set-18*^−/−^*OE SET-18 2#* vs. *set-18*^−/−^) = 0.0026; day 7: ∗∗∗p (*set-18*^−/−^ vs. N2) = 0.0004, ∗p (*set-18*^−/−^*OE SET-18 1#* vs. *set-18*^−/−^) = 0.0195, ∗p (*set-18*^−/−^*OE SET-18 2#* vs. *set-18*^−/−^) = 0.0234; day 11: ∗p (*set-18*^−/−^ vs. N2) = 0.0324, ∗p (*set-18*^−/−^*OE SET-18 1#* vs. *set-18*^−/−^) = 0.0188, ∗p (*set-18*^−/−^*OE SET-18 2#* vs. *set-18*^−/−^) = 0.0242; one-way ANOVA. (D and E) RNAi of *tir-1* and deletion of TIR/SAM domain increased the NAD^+^ level of worms on wild-type background, but not *set-18* mutant one. (D) The NAD^+^ assays of N2 and *set-18* mutant worms with *tir-1* RNAi treatment. The treatment with empty vector was used as negative control. Error bars represent SEM. *n* = 3 biological replicates. ∗∗p (*N2 tir-1 RNAi* vs. *N2 control RNAi*) = 0.0015, p (*set-18*^−/−^*tir-1 RNAi* vs. *set-18*^−/−^*control RNAi*) = 0.5337; Student’s *t* test. (E) The NAD^+^ assays of *tir-1*^*ΔSAM*^ and *tir-1*^*ΔTIR*^ mutants on the background of wild-type (N2) and *set-18* mutant, respectively. Using CRISPR-Cas9 technique, the *set-18*^−/−^*;tir-1*^*ΔSAM*^ and *set-18*^−/−^*;tir-1*^*ΔTIR*^ double mutants were constructed by deleting SAM and TIR domain of TIR-1 in *set-18* mutants, respectively. The NAD^+^ levels were measured and normalized to that of N2. Error bars represent SEM. *n* = 3 biological replicates. ∗∗p (*tir-1*^*ΔSAM*^ vs. N2) = 0.0070, ∗∗∗p (*tir-1*^*ΔTIR*^ vs. N2) < 0.0001, p (*set-18*^−/−^;*tir-1*^*ΔSAM*^ vs. *set-18*^−/−^) = 0.4492, p (*set-18*^−/−^;*tir-1*^*ΔTIR*^ vs. *set-18*^−/−^) = 0.8718; one-way ANOVA. (F) The extended lifespan and increased mtROS-defense ability of *tir-1*^*ΔSAM*^ and *tir-1*^*ΔTIR*^ mutants were both abolished by loss of *set-18*. The worms treated with or without 2 mM PQ were utilized for mtROS-defense assays (+PQ, on the right) and lifespan analysis (on the left), respectively. All of assays were scored based on three independent experiments, with representative examples shown in this figure. Lifespan assays: ∗∗∗p (*tir-1*^*ΔSAM*^ vs. N2) < 0.0001, ∗∗∗p (*tir-1*^*ΔTIR*^ vs. N2) < 0.0001, p (*set-18*^−/−^;*tir-1*^*ΔSAM*^ vs. *set-18*^−/−^) = 0.7992, p (*set-18*^−/−^;*tir-1*^*ΔTIR*^ vs. *set-18*^−/−^) = 0.0739; PQ survival assays: ∗∗∗p (*tir-1*^*ΔSAM*^ vs. N2) < 0.0001, ∗∗∗p (*tir-1*^*ΔTIR*^ vs. N2) < 0.0001, p (*set-18*^−/−^;*tir-1*^*ΔSAM*^ vs. *set-18*^−/−^) = 0.5401, p (*set-18*^−/−^;*tir-1*^*ΔTIR*^ vs. *set-18*^−/−^) = 0.1230; *log rank* test. All data and statistical analyses are provided in Table S3. (G) Mutation of *set-18* alleviated the decrease of mtROS level in *tir-1*^*ΔSAM*^ and *tir-1*^*ΔTIR*^ mutants. Each strain at young (day 3) and old (day 11) ages were treated with/without 2 mM PQ treatment, respectively, and their mtROS levels were analyzed by calculating the ratio of oxidized Grx1-roGFP2 to reduced one in mitochondria. Error bars represent SEM. n = 3–4 biological replicates. Day 3: ∗∗p (*tir-1*^*ΔSAM*^ vs. N2) = 0.0016, ∗∗p (*tir-1*^*ΔTIR*^ vs. N2) = 0.0013, p (*set-18*^−/−^;*tir-1*^*ΔSAM*^ vs. *set-18*^−/−^) = 0.3608, p (*set-18*^−/−^;*tir-1*^*ΔTIR*^ vs. *set-18*^−/−^) = 0.2172; day 3+PQ: ∗∗∗p (*tir-1*^*ΔSAM*^ vs. N2) = 0.0004, ∗∗∗p (*tir-1*^*ΔTIR*^ vs. N2) = 0.0006, p (*set-18*^−/−^;*tir-1*^*ΔSAM*^ vs. *set-18*^−/−^) = 0.7494, p (*set-18*^−/−^;*tir-1*^*ΔTIR*^ vs. *set-18*^−/−^) = 0.0766; day 11: ∗p (*tir-1*^*ΔSAM*^ vs. N2) = 0.0121, ∗∗p (*tir-1*^*ΔTIR*^ vs. N2) = 0.0047, p (*set-18*^−/−^;*tir-1*^*ΔSAM*^ vs. *set-18*^−/−^) = 0.4417, p (*set-18*^−/−^;*tir-1*^*ΔTIR*^ vs. *set-18*^−/−^) = 0.7193; day 11+PQ: ∗∗p (*tir-1*^*ΔSAM*^ vs. N2) = 0.0054, ∗∗p (*tir-1*^*ΔTIR*^ vs. N2) = 0.0046, p (*set-18*^−/−^;*tir-1*^*ΔSAM*^ vs. *set-18*^−/−^) = 0.8284, p (*set-18*^−/−^;*tir-1*^*ΔTIR*^ vs. *set-18*^−/−^) = 0.8418; one-way ANOVA.

To screen the target genes in NAD^+^ metabolic pathway that are controlled by SET-18, we detected the mRNA expression levels of the key NAD^+^ consumption and biosynthesis genes in N2 and *set-18* mutant worms. Results showed that loss of *set-18* decreased the mRNA level of *tir-1*, the homologous of mammalian NAD^+^ hydrolase (NADase) *sterile alpha and TIR motif containing1* (SARM1) (Figure S7), but did not alter the levels of other genes, including nicotinamidase *pnc-1*, NAD^+^ consumer genes *pme-1* and *sir-2.1*, nicotinamide nucleotide adenylyltransferase *nmat-1* and *nmat-2*, NAD^+^ synthase *qns-1* (Figure 3A). We observed that in N2 worms, the mRNA level of *tir-1* gradually increased from day 3 to day 11 (Figure 3B). And through the whole life (day 1, day 3, day 7, and day 11) of worms, loss of *set-18* downregulated the *tir-1* mRNA level, and these declines were all alleviated by rescue of SET-18 expression (Figure 3C). These implied that *tir-1* is a specific target gene of NAD^+^ metabolic pathway activated by SET-18.

It has been reported that TIR-1 is able to catalyze NAD^+^ hydrolysis *in vitro*, and its NADase activity domain (TIR) and self-association domain are both critical for this activity.^41^ However, whether TIR-1 possesses NADase activity *in vivo* remains unclear to date. Here, we found that knockdown of *tir-1* (Figure S8) and deletion of TIR/SAM domain (*tir-1*^*ΔTIR*^ and *tir-1*^*ΔSAM*^) (Figure S9) increased the NAD^+^ level of N2 worms, but not of *set-18* mutants (Figures 3D and 3E). These suggested that TIR-1 functions as a NADase *in vivo* and this enzymatic activity is promoted by SET-18. Additionally, survival assays were conducted, and the result showed that loss of TIR/SAM domain extended the lifespan of N2 worms and increased their ability to defense against PQ-induced mtROS, but such phenotypes shown by the *set-18* mutants were not changed by deletion of these two domains (Figure 3F, Table S3). By analyzing the ratio of oxidized/reduced Grx1-roGFP2 in mitochondria, we observed that the levels of mtROS were declined by deletion TIR/SAM domain in both young (day 3) and old (day 11) wild-type (N2) worms with/without PQ treatment, but did not change in all worms with *set-18* mutation background (Figure 3G). As the offspring of *tir-1*^*ΔSAM*^ and *tir-1*^*ΔTIR*^ mutants that express *P*_*myo-3*_::mito::GFP reporter bear high percentage of wormbags, we failed to detect the influence of TIR/SAM domain on worm’s mitochondrial morphology. Using UPR^mt^ reporter worms (*hsp-6p*::GFP), we found that RNAi of *tir-1* had no impact on UPR^mt^ (Figure S10), as the same as shown by *set-18* mutants. These findings together indicated that SET-18 targets NADase TIR-1 to promote mtROS accumulation-mediated aging.

### SET-18 activates the expression of *tir-1* isoform d in pharynx by histone H3K36me2 modification

**Figure 4:**
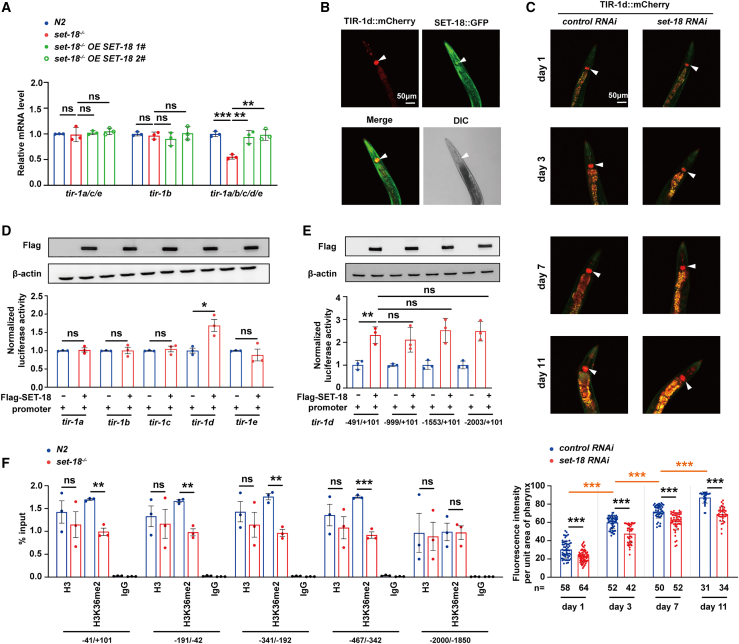
SET-18 activates expression of *tir-1 isoform d* in pharynx by histone H3K36me2 modification (A) The mRNA level of *tir-1* isoform *d* (*tir-1d*) was increased by SET-18. The strains “*set-18*^−/−^*OE SET-18*” *1#* and *2#* were identical to the ones used in Figure 1G. Using isoforms-specific primers, the mRNA levels of *tir-1a/c/e*, *tir-1b*, and *tir-1a/b/c/d/e* in each strain were measured by RT-qPCR and normalized to those of N2 worms. *act-1* was used as an internal reference. Error bars represent SEM. *n* = 3 biological replicates. *tir-1a/c/e*: p (*set-18*^−/−^ vs. N2) = 0.8790, p (*set-18*^−/−^*OE SET-18 1#* vs. *set-18*^−/−^) = 0.6824, p (*set-18*^−/−^*OE SET-18 2#* vs. *set-18*^−/−^) = 0.5140; *tir-1b*: p (*set-18*^−/−^ vs. N2) = 0.5122, p (*set-18*^−/−^*OE SET-18 1#* vs. *set-18*^−/−^) = 0.4875, p (*set-18*^−/−^*OE SET-18 2#* vs. *set-18*^−/−^) = 0.6175; *tir-1a/b/c/d/e*: ∗∗∗p (*set-18*^−/−^ vs. N2) = 0.0004, ∗∗p (*set-18*^−/−^*OE SET-18 1#* vs. *set-18*^−/−^) = 0.0072, ∗∗p (*set-18*^−/−^*OE SET-18 2#* vs. *set-18*^−/−^) = 0.0031; one-way ANOVA. (B) The expressions of TIR-1d::mCherry and SET-18::GFP were mainly co-localized in worm pharynx. The plasmids *P*_*tir-1d*_::TIR-1d::mCherry were co-injected with *rol-6* marker (pRF4) into *tir-1*^*ΔTIR*^ mutants on SET-18::GFP background. The co-localization of TIR-1d::mCherry and SET-18::GFP expression in pharynx (indicated by white arrows) were detected by confocal microscopy, with the representative images shown here. (C) Knockdown of *set-18* decreased TIR-1d::mCherry expression in pharynx during worm aging. The worms with expressing TIR-1d::mCherry were treated with *set-18* RNAi from L1 larva to young (day1 and day 3) and old (day 7 and day 11) ages, respectively. *control RNAi*, empty vector. The TIR-1d::mCherry expressions in the pharynx (indicated by white arrows) of worms were detected by confocal microscopy with the identical exposure settings (the representative images shown in upper). The autofluorescence of worms were shown in orange by using 568 nm in conjunction with 488 nm excitation filters. The fluorescence intensity per unit area of pharynx were then quantified (shown in lower). *n*, the number of worms used for quantitative analysis. Error bars represent SEM. ∗∗∗p (day 3 vs. day 1) < 0.0001, ∗∗∗p (day 7 vs. day 3) < 0.0001, ∗∗∗p (day 11 vs. day 7) < 0.0001; ∗∗∗p (day 1: *set-18*^−/−^ vs. N2) < 0.0001, ∗∗∗p (day 3: *set-18*^−/−^ vs. N2) < 0.0001, ∗∗p (day 7: *set-18*^−/−^ vs. N2) < 0.0001, ∗∗p (day 11: *set-18*^−/−^ vs. N2) < 0.0001; one-way ANOVA and Student’s *t* test. (D) The activity of promoter of *tir-1d*, but not those of other *tir-1* isoforms, was increased by overexpression of SET-18. The luciferase reporter constructs containing the promoters of *tir-1a*, *tir-1b*, *tir-1c*, *tir-1d*, and *tir-1e* were co-transfected with the Flag-tagged SET-18 overexpression plasmids (empty vector was used as the control) in human 293T cells, respectively, followed by luciferase assays. The Flag-SET-18 expression level was confirmed by western blot, using β-actin as an internal reference (shown in upper). The firefly luciferase activity was normalized to groups of control, and renilla was used as the internal reference. Error bars represented SEM. *n* = 3 biological replicates. *Promoter tir-1a*: p (OE SET-18 vs. control) = 0.7219, *Promoter tir-1b*: p (OE SET-18 vs. control) = 0.9367, *Promoter tir-1c*: p (OE SET-18 vs. control) = 0.5702, *Promoter tir-1d*: ∗p (OE SET-18 vs. control) = 0.0175, *Promoter tir-1e*: p (OE SET-18 vs. control) = 0.5112; Student’s *t* test. (E) SET-18 activated the promoter of *tir-1d* within the sequence of −491∼ +101 bp. The luciferase reporter constructs containing the distinct truncated promoters of *tir-1d* within distinct sequences (−2003 ∼ +101 bp, −1553 ∼ +101 bp, −999 ∼ +101 bp, −491 ∼ +101 bp) were co-transfected with Flag-tagged SET-18 overexpression plasmid (empty vector was used as the control) in human 293T cells respectively, followed by luciferase assay. Error bars represented SEM. *n* = 3 biological replicates. ∗∗p (−491 ∼ +101 bp vs. control) = 0.0054, p (−999 ∼ +101 bp vs. −491 ∼ +101) = 0.6232, p (−1553 ∼ +101 bp vs. −491 ∼ +101) = 0.5956, p (−1553 ∼ +101 bp vs. −491 ∼ +101) = 0.6204; one-way ANOVA. (F) Loss of *set-18* decreased H3K36me2 modification level on *tir-1d* promoter. The levels of H3K36me2 modification and histone H3 occupation on the distinct sequences of *tir-1d* promoter within −491 ∼ +101 bp were detected by ChIP-qPCR and presented as percentage of the qPCR signal in total input DNA. The sequence −2000 ∼ −1850 bp was used as a control that was not activated by SET-18. IgG was used as the negative control for ChIP-qPCR. Histone H3 occupation was used as the internal reference. Error bars represent SEM. *n* = 3 biological replicates. −41 ∼ +101 bp: p (H3: *set-18*^*−/−*^ vs. N2) = 0.5013, ∗∗p (H3K36me2: *set-18*^*−/−*^ vs. N2) = 0.0010; −191 ∼ −42 bp: p (H3: *set-18*^*−/−*^ vs. N2) = 0.6900, ∗∗p (H3K36me2: *set-18*^*−/−*^ vs. N2) = 0.0012; −341 ∼ −192 bp: p (H3: *set-18*^*−/−*^ vs. N2) = 0.4620, ∗∗p (H3K36me2: *set-18*^*−/−*^ vs. N2) = 0.0014; −467 ∼ −342 bp: p (H3: *set-18*^*−/−*^ vs. N2) = 0.4584, ∗∗∗p (H3K36me2: *set-18*^*−/−*^ vs. N2) = 0.0003; −341 ∼ −192 bp: p (H3: *set-18*^*−/−*^ vs. N2) = 0.4620, ∗∗p (H3K36me2: *set-18*^*−/−*^ vs. N2) = 0.0014; −2000 ∼ −1850 bp: p (H3: *set-18*^*−/−*^ vs. N2) = 0.8903, p (H3K36me2: *set-18*^*−/−*^ vs. N2) = 0.9553; Student’s *t* test.

There exist five isoforms of *tir-1* genes in *C. elegans*, including *tir-1a*, *tir-1b*, *tir-1c*, *tir-1d*, and *tir-1e* that were predicted to be transcribed by distinct promoters, respectively.^42^ To explore which isoform’s transcription was regulated by SET-18, we designed the isoforms-specific primers for RT-qPCR (Figure S11) and found that loss of *set-18* significantly downregulated the transcripts of *tir-1a/b/c/d/e*, but did not change those of *tir-1b* and *tir-1a/c/e* (Figure 4A). And the decrease of *tir-1a/b/c/d/e* mRNA levels in *set-18* mutants was rescued by overexpression of SET-18 driven by its own promoter (Figure 4A). These data indicated that among the five isoforms, *tir-1d* is primarily activated by SET-18. To detect the expression pattern of TIR-1d in worms, the transgenic worms expressing TIR-1dm::Cherry driven by *tir-1d* own promoter were constructed on *tir-1*^*ΔTIR*^ mutant background. Confocal microscopy and fluorescence intensity analysis showed that TIR-1dm::Cherry expression was enriched in the pharynx where it was colocalized with SET-18::GFP expression (Figures 4B, S12). From young (day 1 and day 3) to old ages (day 7 and day 11), the expression level of TIR-1d::mCherry in the unit area of N2 worms’ pharynx gradually increased (Figures 4C, S13). And at each of worm age, this TIR-1dm::Cherry level was declined by knockdown of *set-18* (Figures 4C, S13). These observations indicated that SET-18 promotes the expression of TIR-1d in pharynx during worm aging.

To explore whether the activity of *tir-1d* promoter was able to be upregulated by SET-18, the overexpression plasmid of flag-tagged SET-18 and the luciferase reporter plasmids containing the promoters of each *tir-1* isoforms (Figure S11) were then co-transfected into human HEK293T cell. The luciferase reporter assays showed that overexpression of SET-18 activated the promoter of *tir-1d*, but not that of *tir-1a*, *tir-1b*, *tir-1c*, and *tir-1e* (Figure 4D**)**. And the expressions of the reporter genes driven by the distinct truncated promoters of *tir-1d* (including the sequence of −2003 ∼ +101 bp, −1553 ∼ +101 bp, −999 ∼ +101 bp, −491 ∼ +101 bp) were all enhanced by SET-18 overexpression at the same level (Figure 4E**)**, indicating that SET-18 activates the *tir-1d* promoter within the sequence of −491 ∼ +101 bp.

Our previous work has shown that *C. elegans* SET-18 is responsible for catalyzing histone H3K36me2 modification, but not H3K4 methylation.^22^ Therefore, four pairs of primers on *tir-1d* promoter (−491 bp ∼ +101 bp) were then designed for histone H3K36me2 ChIP-qPCR in N2 worms and *set-18* mutants. The results showed that loss of *set-18* significantly decreased the H3K36me2 level on the *tir-1d* promoter (Figure 4F). As control, no change of the H3 occupation on this promoter displayed in *set-18* mutants, compared to N2 worms (Figure 4F). These data suggested that SET-18 activate *tir-1d* promoter by histone H3K36me2 modification.

### SET-18-mediated upregulation of TIR-1d promotes aging by increasing mtROS

**Figure 5:**
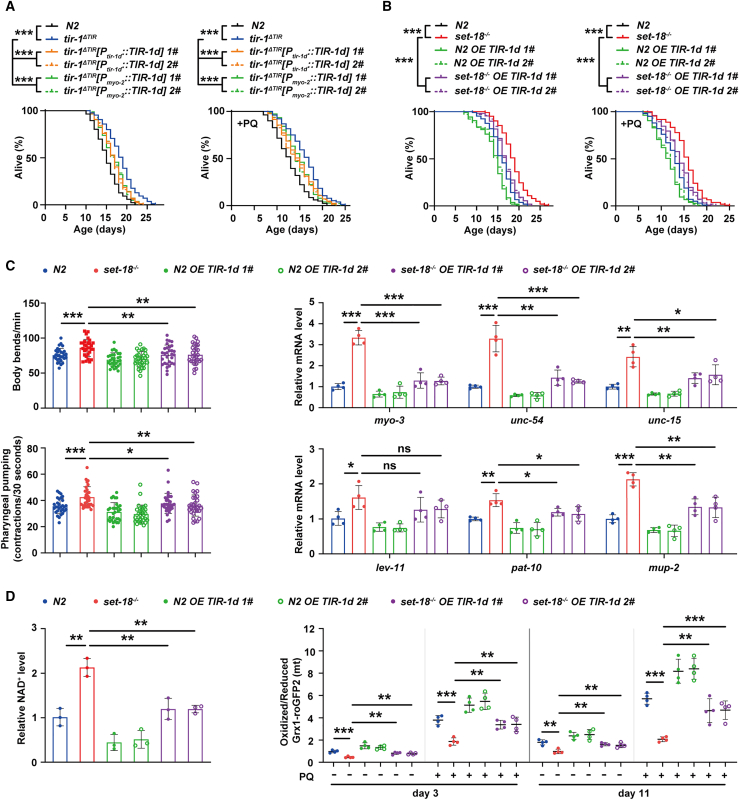
SET-18-mediated TIR-1d activation decreases NAD^+^ level to promote mtROS accumulation-induced aging (A) Rescue of TIR-1d in pharynx attenuated the extended lifespan and enhanced mtROS-defense ability of *tir-1* mutants. The plasmids *P*_*tir-1d*_::TIR-1d::mCherry and *P*_*myo-2*_::TIR-1d::mCherry were, respectively, co-injected with *rol-6* marker (pRF4) into *tir-1*^*ΔTIR*^ mutants to rescue TIR-1d expression driven by its own promoter and pharynx-specific *myo-2* promoter. *1#* and *2#* were two independent extrachromosomal arrays. Here showed the representative examples of lifespan assays (on the left) and mtROS-defense assays (+PQ, on the right). Lifespan assays: ∗∗∗p (*tir-1*^*ΔTIR*^ vs. N2) < 0.0001, ∗∗∗p (*tir-1*^*ΔTIR*^*[P*_*tir-1d*_*::TIR-1d] 1# and 2#* vs. *tir-1*^*ΔTIR*^) < 0.0001, ∗∗∗p (*tir-1*^*ΔTIR*^*[P*_*myo-2*_*::TIR-1d] 1# and 2#* vs. *tir-1*^*ΔTIR*^) < 0.0001; PQ survival assays: ∗∗∗p (*tir-1*^*ΔTIR*^ vs. N2) < 0.0001, ∗∗∗p (*tir-1*^*ΔTIR*^*[P*_*tir-1d*_*::TIR-1d] 1# and 2#* vs. *tir-1*^*ΔTIR*^) < 0.0001, ∗∗∗p (*tir-1*^*ΔTIR*^*[P*_*myo-2*_*::TIR-1d] 1# and 2#* vs. *tir-1*^*ΔTIR*^) < 0.0001; *log rank* test. All data and statistical analyses based on three independent experiments were provided in Table S4. (B) Overexpression of TIR-1d alleviated the lifespan extension and the increase in mtROS-defense ability of *set-18* mutants. “*N2 OE TIR-1d*” and “*set-18*^−/−^*OE TIR-1d*” were obtained by co-injecting plasmid *P*_*tir-1d*_::TIR-1d::mCherry with *rol-6* marker (pRF4) to N2 worm and *set-18* mutants, respectively. *1#* and *2#* were two independent extrachromosomal arrays. Here showed the representative examples of lifespan assays (on the left) and mtROS-defense assays (+PQ, on the right). Lifespan assays: ∗∗∗p (*set-18*^−/−^ vs. N2) < 0.0001, ∗∗∗p (*set-18*^−/−^*OE TIR-1d 1# and 2#* vs. *set-18*^−/−^) < 0.0001, ∗∗∗p (*set-18*^−/−^*OE TIR-1d 1# and 2#* vs. *set-18*^−/−^) < 0.0001; PQ survival assays: ∗∗∗p (*set-18*^−/−^ vs. N2) < 0.0001, ∗∗∗p (*set-18*^−/−^*OE TIR-1d 1# and 2#* vs. *set-18*^−/−^) < 0.0001, ∗∗∗p (*set-18*^−/−^*OE TIR-1d 1# and 2#* vs. *set-18*^−/−^) < 0.0001; *log rank* test. All data and statistical analyses based on three independent experiments were provided in Table S5. (C) The enhanced muscle functions of *set-18* mutants at old age (day 11) were abolished by overexpression of TIR-1d. The strains “*N2 OE TIR-1d*” and “*set-18*^−/−^*OE TIR-1d*” *1#* and *2#* were identical to the ones used in Figure 5B. The frequency of body bend was represented by the average numbers of worm body bends during 1 min (upper, on the left). The rate of pharyngeal pumping was assessed by calculating the number of worm pharyngeal contractions during 30 s (lower, on the left). The mRNA levels of genes encoding the muscle proteins were measured by RT-qPCR and normalized to those of N2 (on the right). *act-1* was used as an internal reference. Error bars represent SEM. Body bending frequency: *n* = 30 worms per group. ∗∗∗p (*set-18*^−/−^ vs. N2) = 0.0005, ∗∗p (*set-18*^−/−^*OE TIR-1d 1#* vs. *set-18*^−/−^) = 0.0042, ∗∗p (*set-18*^−/−^*OE TIR-1d 2#* vs. *set-18*^−/−^) = 0.0043; pharyngeal pump rate: *n* = 30 worms per group. ∗∗∗p (*set-18*^−/−^ vs. N2) = 0.0003, ∗p (*set-18*^−/−^*OE TIR-1d 1#* vs. *set-18*^−/−^) = 0.0226, ∗∗p (*set-18*^−/−^*OE TIR-1d 2#* vs. *set-18*^−/−^) = 0.0031; RT-qPCR: *n* = 3 biological replicates. *myo-3*: ∗∗∗p (*set-18*^−/−^ vs. N2) < 0.0001, ∗∗∗p (*set-18*^−/−^*OE TIR-1d 1#* vs. *set-18*^−/−^) = 0.0002, ∗∗p (*set-18*^−/−^*OE TIR-1d 2#* vs. *set-18*^−/−^) < 0.0001; *unc-54*:∗∗∗p (*set-18*^−/−^ vs. N2) = 0.0003, ∗∗p (*set-18*^−/−^*OE TIR-1d 1#* vs. *set-18*^−/−^) = 0.0022, ∗∗∗p (*set-18*^−/−^*OE TIR-1d 2#* vs. *set-18*^−/−^) = 0.0007; *unc-15*: ∗∗∗p (*set-18*^−/−^ vs. N2) = 0.0010, ∗∗p (*set-18*^−/−^*OE TIR-1d 1#* vs. *set-18*^−/−^) = 0.0095, ∗p (*set-18*^−/−^*OE TIR-1d 2#* vs. *set-18*^−/−^) = 0.0438; *lev-11*: ∗p (*set-18*^−/−^ vs. N2) = 0.0115, p (*set-18*^−/−^*OE TIR-1d 1#* vs. *set-18*^−/−^) = 0.2067, ∗p (*set-18*^−/−^*OE TIR-1d 2#* vs. *set-18*^−/−^) = 0.1789; *pat-10*: ∗∗p (*set-18*^−/−^ vs. N2) = 0.0011, ∗p (*set-18*^−/−^*OE TIR-1d 1#* vs. *set-18*^−/−^) = 0.0188, ∗p (*set-18*^−/−^*OE TIR-1d 2#* vs. *set-18*^−/−^) = 0.0299; *mup-2*: ∗∗∗p (*set-18*^−/−^ vs. N2) < 0.0001, ∗∗p (*set-18*^−/−^*OE TIR-1d 1#* vs. *set-18*^−/−^) = 0.0018, ∗∗p (*set-18*^−/−^*OE TIR-1d 2#* vs. *set-18*^−/−^) = 0.0037; one-way ANOVA. All data and statistical analyses were provided in Table S6. (D) The increased NAD^+^ level and reduced mtROS accumulation in *set-18* mutants were both attenuated by overexpression of TIR-1d. The strains “*N2 OE TIR-1d*” and “*set-18*^−/−^*OE TIR-1d*” *1#* and *2#* were identical to the ones used in Figure 5B. The NAD^+^ levels were detected by microplate reader and normalized to that of N2 (shown on the left). The worms at young (day 3) and old (day 11) ages were treated with/without 2 mM PQ respectively, and their mtROS levels were analyzed by calculating the ratio of oxidized Grx1-roGFP2 to reduced one in mitochondria (shown on the right). Error bars represent SEM. NAD^+^ level: *n* = 3 biological replicates. ∗∗p (*set-18*^−/−^ vs. N2) = 0.0023, ∗∗p (*set-18*^−/−^*OE TIR-1d 1#* vs. *set-18*^−/−^) = 0.0067, ∗∗p (*set-18*^−/−^*OE TIR-1d 2#* vs. *set-18*^−/−^) = 0.0018; one-way ANOVA. mtROS level: *n* = 4 biological replicates. Day 3: ∗∗∗p (*set-18*^−/−^ vs. N2) = 0.0004, ∗∗p (*set-18*^−/−^*OE TIR-1d 1#* vs. *set-18*^−/−^) = 0.0017, ∗∗p (*set-18*^−/−^*OE TIR-1d 2#* vs. *set-18*^−/−^) = 0.0012; Day 3+PQ: ∗∗∗p (*set-18*^−/−^ vs. N2) = 0.0003, ∗∗p (*set-18*^−/−^*OE TIR-1d 1#* vs. *set-18*^−/−^) = 0.0011, ∗∗p (*set-18*^−/−^*OE TIR-1d 2#* vs. *set-18*^−/−^) = 0.0055; Day 11: ∗∗p (*set-18*^−/−^ vs. N2) = 0.0018, ∗∗p (*set-18*^−/−^*OE TIR-1d 1#* vs. *set-18*^−/−^) = 0.0021, ∗∗p (*set-18*^−/−^*OE TIR-1d 2#* vs. *set-18*^−/−^) = 0.0097; Day 11+PQ: ∗∗∗p (*set-18*^−/−^ vs. N2) < 0.0001, ∗∗p (*set-18*^−/−^*OE TIR-1d 1#* vs. *set-18*^−/−^) = 0.0033, ∗∗∗p (*set-18*^−/−^*OE TIR-1d 2#* vs. *set-18*^−/−^) = 0.0009; one-way ANOVA.

To confirm whether the specific expression of *tir-1d* in pharynx is able to increase worm aging, we constructed the *P*_*myo-2*_::TIR-1d::mCherry transgenic worms to rescue TIR-1d expression driven by pharynx-specific *myo-2* promoter in *tir-1*^*ΔTIR*^ mutants. We found that specific rescue of TIR-1d expression in pharynx alleviated the lifespan extension and the increase in defense against PQ-induced mtROS in *tir-1*^*ΔTIR*^ mutants, the phenotypes as same as shown by the mutants resuming the expression of TIR-1d driven by its own promoter (*P*_*tir-1d*_::TIR-1d::mCherry) (Figure 5A, Table S4). These observations indicated that the specific expression of *tir-1d* in pharynx contributes to promoting worm aging.

As the exon sequences of *tir-1d* are contained in other isoforms (Figure S11), it is not feasible to conduct the RNAi that specifically targeting *tir-1d* in the worms with *set-18* overexpression. Therefore, to further explore whether SET-18 accelerates aging through upregulating TIR-1d expression, we used transgenic technique to overexpress *P*_*tir-1d*_::TIR-1d::mCherry (OE TIR-1d) in *set-18* mutants and N2 worms, respectively, since our results showed that expression of TIR-1d in pharynx decreased in *set-18* mutants. Survival assays showed that overexpression of TIR-1d attenuated the extended lifespan and enhanced ability of *set-18* mutants to defense against PQ-induced mtROS (Figures 5B, Table S5). The locomotor ability of these worms were also detected as the decline of muscle function is identified as a typical mark of aging conserved from worms to mammals. The results showed that loss of *set-18* improved the locomotor ability of the worms at old age (day 11), including increasing the frequency of body bend (Figures 5C, Table S6) and elevating the rate of pharyngeal pumping (Figure 5C, Table S6). Moreover, mutation of *set-18* enhanced the mRNA levels of some muscle-protein-encoding genes, such as the genes of myosin heavy chains *myo-3* and *unc-54*, paramyosin *unc-15*, tropomyosin *lev-1*, and the subunits of troponin complex *pat-10* and *mup-2* (Figure 5C). However, these improved phenotypes in *set-18* mutants were all attenuated by overexpression of TIR-1d (Figure 5C, Table S6). These combined data suggested that SET-18-mediated upregulation of TIR-1d promotes worm aging.

Moreover, NAD^+^ assays showed that overexpression of TIR-1d abolished the upregulated NAD^+^ level in *set-18* mutants (Figure 5D). By analyzing the ratio of oxidized/reduced Grx1-roGFP2 in mitochondria, we found that the decrease in mtROS levels of *set-18* mutants at young (day 3) and old (day 11) ages were both attenuated by overexpression of TIR-1d, no matter whether they were treated with PQ or not (Figure 5D). Collectively, these findings indicated that SET-18-mediated increase in TIR-1d expression is responsible for the decrease in NAD^+^ level, thereby promoting *C. elegans* aging through enhancing mtROS accumulation.

### SMYD2-mediated activation of SARM1 expression accelerates muscle atrophy via enhancing mtROS

**Figure 6:**
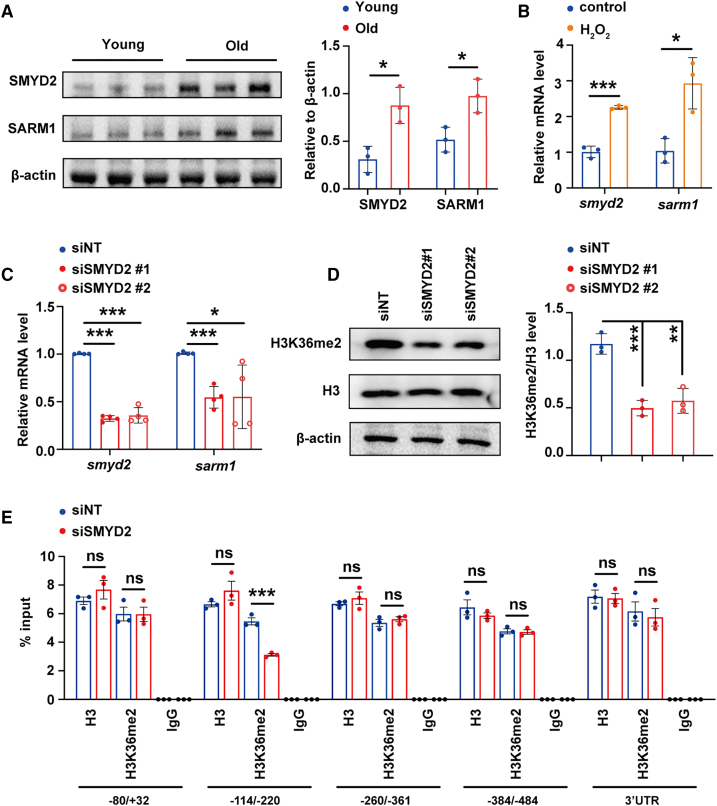
SMYD2 activates the expression of SARM1 by histone H3K36me2 modification during mammalian muscle atrophy (A) The protein levels of SMYD2 and SARM1 were both increased in the muscles of aged mice, compared to the young ones. The protein levels of SMYD2 and SARM1 in the muscles of C57BL/6J mice at old (20 months) and young (6–8 weeks) ages were detected by Western blot (on the left) and quantified by densitometry analysis based on three independent replicates (on the right). β-actin was used as an internal reference. Error bars represent SEM. *n* = 3 mice per group. SMYD2: ∗p (old vs. young) = 0.0140, SARM1: ∗p (old vs. young) = 0.0224; *Student’s t* test. (B) The mRNA levels of *smyd2* and *sarm1* in C2C12 myotubes were raised by H_2_O_2_ treatment. To build an *in vitro* model of muscle atrophy, the differentiated C2C12 myotubes were treated with 700 μM H_2_O_2_ for 24 h. In control, double distilled water instead of H_2_O_2_. The mRNA levels of *smyd2* and *sarm1* were measured by RT-qPCR and normalized to those of control. *β-actin* was used as an internal reference. Error bars represent SEM. *n* = 3 biological replicates. *Smyd2*:∗∗∗p (H_2_O_2_ vs. control) = 0.0002, *sarm1*:∗p (H_2_O_2_ vs. control) = 0.0146; *Student’s t* test. (C) The *sarm1* mRNA level of C2C12 myotubes was downregulated by RNAi of *smyd2*. In Figures 6C–6E, RNAi was performed by transfecting *smyd2* siRNA (siSMYD2) into the differentiated C2C12 myotubes. *#1* and *#2* were two of siRNAs targeting the distinct coding sequences of *smyd2*. The non-targeting siRNA (siNT) was used as control. The mRNA levels of *smyd2* and *sarm1* were measured by RT-qPCR and normalized to those of control. *β-actin* was used as an internal reference. Error bars represent SEM. *n* = 3 biological replicates. *Smyd2*: ∗∗∗p (siSMYD2 #1 vs. siNT) < 0.0001, ∗∗∗p (siSMYD2 #2 vs. siNT) < 0.0001; *sarm1*: ∗∗∗p (siSMYD2 #1 vs. siNT) = 0.0002, ∗∗∗p (siSMYD2 #2 vs. siNT) = 0.0335; one-way ANOVA. (D) Knockdown of *smyd2* reduced the global histone H3K36me2 modification level in C2C12 myotubes. The global H3K36me2 modification levels were detected by Western blot (on the left), and then quantified by densitometry analysis and normalized to those of control based on three independent replicates (on the right). Histone H3 was used as an internal reference. Error bars represented SEM. *n* = 3 biological replicates. ∗∗∗p (siSMYD2 #1 vs. siNT) = 0.0010, ∗∗p (siSMYD2 #2 vs. siNT) = 0.0036; one-way ANOVA. (E) The H3K36me2 modification level on *sarm1* promoter was decreased by RNAi of *smyd2*. The siSMYD2 *#1* was used in this figure. The levels of H3K36me2 modification and histone H3 occupation on the distinct sequences of *sarm1* promoter within −484 bp ∼ +32 bp were detected by ChIP-qPCR and presented as percentage of the qPCR signal in total input DNA. IgG was used as the negative control. H3 occupation was used as the internal reference. Error bars represent SEM. *n* = 3 biological replicates. −80 ∼ +32 bp: p (H3: siSMYD2 vs. siNT) = 0.3348, p (H3K36me2: siSMYD2 vs. siNT) = 0.9800; −114 ∼ −220 bp: p (H3: siSMYD2 vs. siNT) = 0.2327, ∗∗∗p (H3K36me2: siSMYD2 vs. siNT) = 0.0008; −260 ∼ −361 bp: p (H3: siSMYD2 vs. siNT) = 0.4380, p (H3K36me2: siSMYD2 vs. siNT) = 0.4472; −384 ∼ −484 bp: p (H3: siSMYD2 vs. siNT) = 0.3556, p (H3K36me2: siSMYD2 vs. siNT) = 0.8456; 3′UTR: p (H3: siSMYD2 vs. siNT) = 0.8392, p (H3K36me2: siSMYD2 vs. siNT) = 0.6748; Student’s *t* test.

Mammalian histone H3K36me2 methyltransferase SMYD2 and NADase SARM1 are the homologous of *C. elegans* SET-18 and TIR-1, respectively; and they have been reported to play key roles in modulating muscle development or atrophy in mammals.^16^^,^^43^ We found that the protein expression levels of both SMYD2 and SARM1 significantly increased in the muscle of aged mice (20 months), compared to the young mice (6–8 weeks) (Figure 6A). Muscle atrophy is a typical feature of aging.^44^^,^^45^ Therefore, we built an *in vitro* model of muscle atrophy by treating C2C12 myoblast-derived myotubes with H_2_O_2_ according to previous publications.^46^^,^^47^ We observed that the mRNA levels of *smyd2* and *sarm1* both raised in the H_2_O_2_-treated myotubes (Figure 6B), which expression tendency was similar to the enhanced expressions of SET-18 and TIR-1 in the older worms. These results together inspire us to suppose that SMYD2 might promote muscle atrophy by upregulating SARM1 expression, which mechanism is conserved to that SET-18 activates the expression of TIR-1d to accelerate worm aging.

RT-qPCR results showed that *symd2* siRNA decreased the mRNA level of *sarm1* in C2C12 myotubes (Figure 6C). And knockdown of *symd2* also declined the global histone H3K36me2 modification level (Figure 6D), which was consistent to previous reports.^48^^,^^49^ According to the site of *tir-1d* promoter where H3K36me2 modification level was decreased by loss of *set-18*, we designed four pairs of primers for the H3K36me2 ChIP-qPCR on the *sarm1* promoter within the sequence −484 bp ∼ +32 bp. ChIP-qPCR results showed that *symd2* siRNA decreased the H3K36me2 level on the sequence (−220 bp ∼ −114 bp) of *sarm1* promoter, but did not change the H3 occupation on this site (Figure 6E). These findings indicated that SARM1/TIR-1d expressions are conservatively activated by SMYD2/SET-18-mediated histone H3K36me2 modification.

**Figure 7:**
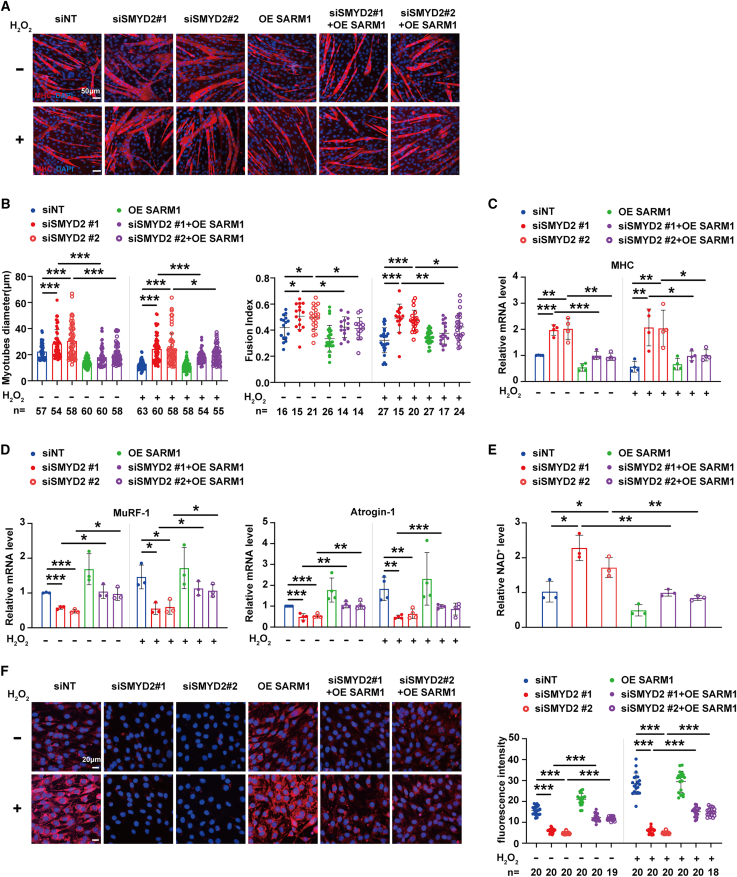
SMYD2-mediated activation of SARM1 reduces NAD^+^ level to promote C2C12 myotube atrophy via increase of mtROS (A and B) The effect of *smyd2* RNAi in reducing C2C12 myotubes atrophy was abolished by overexpression of SARM1. In Figures 7A–7F, *smyd2* siRNA (siSMYD2) was co-transfected with/without the overexpression plasmid of *sarm1* (OE SARM1) into the differentiated C2C12 myotubes. *#1* and *#2* were two of siRNAs targeting the distinct coding sequences of *smyd2*. The non-targeting siRNA (siNT) was used as control. The transfected myotubes were then treated with or without H_2_O_2_, respectively. (A) The representative images of C2C12 myotubes with MHC staining. Red, MHC protein; Blue, the nuclei stained by DAPI. (B) The analysis of myotube diameter and fusion index. The fusion index was indicated as the value of the number of nuclei present in myotubes divided by the number of total nuclei in MHC^+^ cells. Error bars represent SEM. Myotube diameter: without H_2_O_2_: ∗∗∗p (siSMYD2 #1 vs. siNT) = 0.0003, ∗∗∗p (siSMYD2 #2 vs. siNT) < 0.0001, ∗∗∗p (siSMYD2 #1+OE SARM1 vs. siSMYD2 #1) < 0.0001, ∗∗∗p (siSMYD2 #2+OE SARM1 vs. siSMYD2 #2) < 0.0001; with H_2_O_2_: ∗∗∗p (siSMYD2 #1 vs. siNT) < 0.0001, ∗∗∗p (siSMYD2 #2 vs. siNT) < 0.0001, ∗∗∗p (siSMYD2 #1+OE SARM1 vs. siSMYD2 #1) < 0.0001, ∗p (siSMYD2 #2+OE SARM1 vs. siSMYD2 #2) = 0.0365; Fusion index: without H_2_O_2_: ∗p (siSMYD2 #1 vs. siNT) = 0.0162, ∗p (siSMYD2 #2 vs. siNT) = 0.0171, ∗p (siSMYD2 #1+OE SARM1 vs. siSMYD2 #1) = 0.0191, ∗p (siSMYD2 #2+OE SARM1 vs. siSMYD2 #2) = 0.0101; with H_2_O_2_: ∗∗∗p (siSMYD2 #1 vs. siNT) < 0.0001, ∗∗∗p (siSMYD2 #2 vs. siNT) < 0.0001, ∗∗p (siSMYD2 #1+OE SARM1 vs. siSMYD2 #1) = 0.0017, ∗p (siSMYD2 #2+OE SARM1 vs. siSMYD2 #2) = 0.0449; one-way ANOVA. (C and D) Overexpression of SARM1 attenuated the influences of *smyd2* RNAi in up-regulating MHC mRNA level and downregulating the mRNA levels of atrophy-related genes in C2C12 myotubes with/without H_2_O_2_ treatments. The mRNA levels of *MHC* (C), *MuRF1* and *Atrogin-1* (D) in myotubes were measured by RT-qPCR and normalized to those of control groups without H_2_O_2_ treatment. *β-actin* was used as an internal reference. Error bars represent SEM. n = 3–4 biological replicates. *MHC* mRNA level: without H_2_O_2_: ∗∗∗p (siSMYD2 #1 vs. siNT) < 0.0001, ∗∗p (siSMYD2 #2 vs. siNT) = 0.0025, ∗∗∗p (siSMYD2 #1+OE SARM1 vs. siSMYD2 #1) = 0.0003, ∗∗p (siSMYD2 #2+OE SARM1 vs. siSMYD2 #2) = 0.0025; with H_2_O_2_: ∗∗p (siSMYD2 #1 vs. siNT) = 0.0065, ∗∗p (siSMYD2 #2 vs. siNT) = 0.0069, ∗p (siSMYD2 #1+OE SARM1 vs. siSMYD2 #1) = 0.0246, ∗p (siSMYD2 #2+OE SARM1 vs. siSMYD2 #2) = 0.0306; *MuRF1* mRNA level: without H_2_O_2_: ∗∗∗p (siSMYD2 #1 vs. siNT) = 0.0001, ∗∗∗p (siSMYD2 #2 vs. siNT) < 0.0001, ∗p (siSMYD2 #1+OE SARM1 vs. siSMYD2 #1) = 0.0172, ∗p (siSMYD2 #2+OE SARM1 vs. siSMYD2 #2) = 0.0111; with H_2_O_2_: ∗p (siSMYD2 #1 vs. siNT) = 0.0140, ∗p (siSMYD2 #2 vs. siNT) = 0.0196, ∗p (siSMYD2 #1+OE SARM1 vs. siSMYD2 #1) = 0.0189, ∗p (siSMYD2 #2+OE SARM1 vs. siSMYD2 #2) = 0.0407; *Atrogin-1* mRNA level: without H_2_O_2_: ∗∗∗p (siSMYD2 #1 vs. siNT) = 0.0006, ∗∗∗p (siSMYD2 #2 vs. siNT) < 0.0001, ∗∗p (siSMYD2 #1+OE SARM1 vs. siSMYD2 #1) = 0.0018, ∗∗p (siSMYD2 #2+OE SARM1 vs. siSMYD2 #2) = 0.0030; with H_2_O_2_: ∗∗p (siSMYD2 #1 vs. siNT) = 0.0031, ∗∗p (siSMYD2 #2 vs. siNT) = 0.0073, ∗∗∗p (siSMYD2 #1+OE SARM1 vs. siSMYD2 #1) = 0.0002; one-way ANOVA. (E) Overexpression of SARM1 attenuated the increased NAD^+^ level of C2C12 myotubes with *smyd2* RNAi treatment. The NAD^+^ levels of myotubes were detected with microplate reader and normalized to those of control without H_2_O_2_ treatment. Error bars represent SEM. *n* = 3 biological replicates. ∗p (siSMYD2 #1 vs. siNT) = 0.0100, ∗p (siSMYD2 #2 vs. siNT) = 0.0451, ∗∗p (siSMYD2 #1+OE SARM1 vs. siSMYD2 #1) = 0.0041, ∗∗p (siSMYD2 #2+OE SARM1 vs. siSMYD2 #2) = 0.0069; one-way ANOVA. (F) Overexpression of SARM1 alleviated the effect of *smyd2* RNAi in decreasing mtROS level in C2C12 myotubes with/without H_2_O_2_ treatments. The mtROS and nuclei (Blue) in myotubes were stained with MitoSOX and DAPI, respectively. The stained myotubes were detected by confocal microscopy (the representative images showed on the left), followed by quantitative analysis of fluorescence intensity (on the right). Error bars represent SEM. Without H_2_O_2_: ∗∗∗p (siSMYD2 #1 vs. siNT) < 0.0001, ∗∗∗p (siSMYD2 #2 vs. siNT) < 0.0001, ∗∗∗p (siSMYD2 #1+OE SARM1 vs. siSMYD2 #1) < 0.0001, ∗∗∗p (siSMYD2 #2+OE SARM1 vs. siSMYD2 #2) < 0.0001; with H_2_O_2_: ∗∗∗p (siSMYD2 #1 vs. siNT) < 0.0001, ∗∗∗p (siSMYD2 #2 vs. siNT) < 0.0001, ∗∗∗p (siSMYD2 #1+OE SARM1 vs. siSMYD2 #1) < 0.0001, ∗∗∗p (siSMYD2 #2+OE SARM1 vs. siSMYD2 #2) < 0.0001; one-way ANOVA.

To further explore whether SMYD2-mediated activation of SARM1 is able to decrease NAD^+^ level to accelerate mtROS accumulation-induced muscle atrophy, SARM1 was overexpressed (OE SARM1) in the C2C12 myotubes with/without *symd2* siRNA treatments. The result of immunofluorescence staining of MHC showed that no matter whether the C2C12 myotubes were treated with or without H_2_O_2_, knockdown of *smyd2* enlarged the myotube diameter and increased their fusion index (Figures 7A and 7B), and both of these two phenotypes were attenuated by overexpression of SARM1 (Figures 7A and 7B). RT-qPCR analysis showed that rescue of SARM1 alleviated the effects of *symd2* siRNA treatments in upregulating the mRNA level of *MHC* (Figure 7C**)** and downregulating the mRNA levels of atrophy-related genes *MuRF1*and *Atrogin-1* (Figure 7D). These implied that SMYD2-mediated activation of SARM1 increases the natural or H_2_O_2_-induced muscle atrophy in mouse.

Moreover, NAD^+^ assays showed that knockdown of *smyd2* raised the NAD^+^ level in C2C12 myotubes, and this increase was attenuated by overexpression of SARM1 (OE SARM1) (Figure 7E). Using mitoSOX to detect mtROS level, we observed that overexpression of SARM1 alleviated the *smyd2* siRNA-induced decrease in mtROS level in C2C12 myotubes with/without H_2_O_2_ treatment (Figure 7F). These findings together suggested that SMYD2-mediated activation of SARM1 reduces NAD^+^ level to accelerate the mtROS accumulation-induced muscle atrophy, a regulatory mechanism conserved with that SET-18-mediated activation of TIR-1d increases *C. elegans* aging.

## Discussion

NAD^+^ is a critical coenzyme for redox reactions and also an essential cofactor for non-redox NAD^+^-dependent enzymes (including NADase, Sirtuins, and PARPs), making it central to various cellular and biological processes.^50^ The close link between NAD^+^ level and aging has been established for decades.^51^ In human and multiple model organisms, a gradual decline of NAD^+^ levels in cells and tissues is a typical phenomenon accompanying aging.^52^ Upregulation of NADases expression has been identified as a crucial reason of this decrease of NAD^+^ levels^53^; however, the molecular mechanisms interpreting how NADase expression is controlled remain poorly understood. In this study, we discovered that during aging, histone methyltransferase SET-18/SMYD2-mediated H3K36me2 modification promotes the expression of NADase gene TIR-1d/SARM1, causing an increase in mtROS accumulation in *C. elegans* and mammal. These findings suggest that “H3K36me2-NADase-mtROS” is a novel “nucleus-to-mitochondria” anterograde communication that plays a role in aging control and is conserved from worms to mammals.

The accumulation of mtROS during aging is generally considered a passive result of increased ETC leakage.^54^ However, our present results showed that mtROS level was actively upregulated by SET-18/SMYD2-mediated activation of TIR-1d/SARM1 expression, implying that the increase of mtROS production accompanying aging is a procedurally-controlled process. It is well-known that the mitochondrial stress signalings (including UPR^mt^ and mtROS) modulate aging by driving epigenetic regulation, which is referred as a “mitochondria-to-nucleus” communication.^55^ Here, we found that H_2_O_2_ treatment enhanced mtROS level in C2C12 myotubes, and also activated the expression of SMYD2 (Figure 6) and raised the global level of H3K36me2 modification (data not shown). And our previous report showed that the levels of both SET-18 expression and histone H3K36me2 modification increased during worm aging,^22^ companied by the increase of mtROS production (Figure 1). These data inspire us to speculate that while mtROS accumulation is elevated by histone H3K36me2 methyltransferase SET-18/SMYD2, this increased mtROS level might then be enhanced via a positive feedback of increasing SET-18/SMYD2-mediated H3K36me2 modification, which is supposed that aging is programmed by the “H3K36me2-mtROS” bidirectional communications between nucleus and mitochondria. Although ROS is considered to be “toxic” to promote aging, it also displays “hormesis” as that increased ROS at low-dosage or specific developmental stages result in lifespan extension.^31^^,^^56^ It is reported that the higher ROS level at L2 larval stage of worms, compared to that of adults, contributes to increased lifespan.^31^ Therefore, it will be interesting to further explore whether and what kind of epigenetic modifications drive this ROS elevation at developmental stage to promote longevity.

The mitochondrial redox is balanced by ETC leakage-mediated mtROS generation and antioxidase-catalyzed mtROS clearance.^57^ Intriguingly, our previous study showed that the expressions of *daf-16a*, the key transcriptional factor responsible for promoting the transcription of several antioxidase genes (such as *gst-20*, *ctl-3*) were inhibited by SET-18-mediated H3K36me2 modification because this modification enhanced the recruitment of a histone deacetylase 1 (HDAC1) homolog on the *daf-16a*′s promoter.^22^ Therefore, these findings together with the present work indicated that SET-18/SMYD2-mediated H3K36me2 modification destroys mitochondrial redox balance through a strategy acting on both aspects of the generation and clearance of mtROS: on the one hand, it increases mtROS production by activating the expression of target gene *tir-1d/sarm1*; and on the other hand, it represses the expression of *daf-16a* to decrease the antioxidases’ activities. Nevertheless, whether the genes of NADH generation and oxidative metabolism that also regulate mitochondrial redox balance, or even the genes in other cellular activities and biological processes, are targeted by SET-18/SMYD2-mediated H3K36me2 modification is worthy to be further explored.

The results of our and others’ have showed that SET-18/SMYD2 downregulate target gene expressions by the crosstalk between H3K36me2 modification and histone deactylation.^22^ However, what kind of histone modification is required for the crosstalk with SET-18/SMYD2-mediated H3K36me2 modification to upregulate target gene expressions remains unclear. Evidence shows that histone H3K36me2 methyltransferase NSD1 and NSD2 increased their target genes’ expressions by attenuating H3K27me3 modification on their promoters.^58^^,^^59^^,^^60^ Therefore, it will be worthy to further explore whether SET-18/SMYD2 promote *tir-1d/sarm1* expressions by the crosstalk between histone H3K36me2 and H3K27me3 modifications. Moreover, given that SMYD2 also catalyzes H3K4 methylation,^61^ whether SMYD2-mediated H3K4me modification modulates novel target genes to promote muscle atrophy is also interesting to be investigated.

There are three NADase genes in mammals, including SARM1, CD38, and CD157. It is reported that expression of SARM1 in neuron contributes to promoting axonal degeneration after injury^62^; CD38 expressed in immune cells plays roles in inflammation and autoimmunity^63^^,^^64^; and high expression of CD157 correlates with the increase of pithelial ovarian tumor aggressiveness.^65^^,^^66^ However, in *C. elegans*, the single NADase gene TIR-1 is transcribed to distinct isoforms driven by different promoters. In this study, we observed that the *tir-1 isoform d* expression was enriched in pharyngeal muscle and it was required for the function of SET-18 in promoting aging. It is reported that the specific expression of *tir-1 isoform b* in GABA motor neurons is able to regulate axon regeneration and axonal degeneration.^67^ These findings together imply that the tissue-specific expressions of distinct *tir-1* isoforms might resemble the various types of NADases in mammals. In addition, overexpression or knockdown the genes in the specific tissues may regulate lifespan in a cell-non-autonomous fashion.^12^^,^^68^ For example, overexpression of histone demethylases *jmjd-1.2* and *jmjd-3.1* and knocking down the genes of ETC components specifically in *C. elegans* nervous system both activated intestinal UPR^mt^ to promote longevity.^12^^,^^68^ Therefore, whether the SET-18-mediated activation of *tir-1d* expression in pharynx might change the NAD^+^ and mtROS levels in other tissues and modulate worm lifespan in a cell-non-autonomous fashion is worth exploring in future.

It is known that the conserved domains of TIR-1/SARM1 include an auto-inhibitory N-terminal ARM domain, the tandem repeat SAM domains that drive protein oligomerization, and a C-terminal TIR domain responsible for catalyzing NAD^+^ hydrolysis.^41^^,^^69^ While ARM domain interacts with TIR domain, oligomerization of TIR-1/SARM1 is prevented, thereby disrupting its NADase activity.^69^^,^^70^ Compared with the other isoforms of *tir-1* in worms, TIR-1d lacks the ARM domain,^42^ suggesting that TIR-1d might have stronger NADase activity. And this might be the reason why the SET-18-mediated activation of TIR-1d expression has physiological effect on decreasing the NAD^+^ level during worm aging. Moreover, the NADase activity of *C. elegans* TIR-1 is also documented to regulate innate immunity response via p38/MAPK pathway. Mutation of TIR domain or blocking TIR-1 oligomerization is able to repress p38/PMK-1 phosphorylation during bacterial infection.^60^ And activation of p38/MAPK signaling is benefit for longevity.^71^ Therefore, it will be interesting to further investigate whether p38/MAPK pathway is also required for the effect of SET-18-mediated TIR-1d activation in aging control.

**Figure 8:**
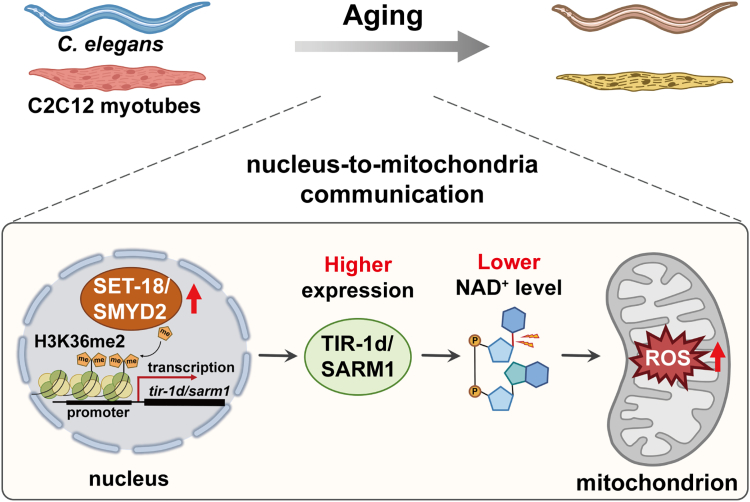
The schematic model illustrates the mechanism that histone H3K36 dimethyltrasferase SET-18/SMYD2-mediated activation of NADase TIR-1d/SARM1 increases mtROS to promote aging, which is conserved from *C. elegans* to mouse

In summary, histone H3K36me2 methyltransferase SET-18/SMYD2 were responsible for activating NADase *tir-1d/sarm1* expressions by histone H3K36me2 modification on their promoters. And this increase of NADase activity promoted hydrolysis of NAD^+^, thereby enhancing mtROS accumulation, and consequently shortening worm lifespan and accelerating mouse myotube atrophy (Figure 8). The present study not only revealed an epigenetic mechanism of controlling the decline of NAD^+^ levels accompanying aging, but also provide a novel target that represses NADase overexpression to potentially extend lifespan and treat aging-related diseases.

### Limitations of the study

This study reveals that histone methyltransferase SET-18/SMYD2-mediated activation of NADase TIR-1d/SARM1 increases mtROS, thereby accelerating aging in both *Caenorhabditis elegans* and mouse muscle tissues. However, whether this mechanism accounts for mouse lifespan extension remains to be verified. While histone lysine methylation typically influences multiple gene expressions, this research specifically focuses on SET-18/SMYD2’s role in NAD^+^ metabolism. Therefore, RNA sequencing analysis is required to identify additional target genes and signaling pathways regulated by SET-18/SMYD2. Moreover, since the *tir-1d* transcript is nested within other *tir-1* subtypes’ transcripts, we were unable to directly detect *tir-1d* transcription or perform *tir-1d*-specific RNA interference or gene knockout experiments in SET-18 overexpressed nematodes.

## Resource availability

### Lead contact

Requests for further information and resources should be directed to and will be fulfilled by the lead contact, Xiaoxue Li (lixx956@nenu.edu.cn).

### Materials availability

All materials generated in this study are available from the lead contact.

### Data and code availability

• Data reported in this paper will be shared by the lead contact upon request.
• This paper does not report original code.
• Any additional information required to reanalyze the data reported in this paper are available from the lead contact upon request.

## Acknowledgments

This work was supported by the 10.13039/100014718National Natural Science Foundation of China (no. 32371323) and the 10.13039/100017369Scientific and Technologic Foundation of Jilin Province (no. 20250102265JC). We thank Prof. Chang Chen (Institutes of Biophysics, Chinese Academy of Sciences) for providing worm strains with Grx1-roGFP2 reporters. We frankly thank all participants involved in this study, finally.

## Author contributions

Resources, data curation, software, formal analysis, validation, investigation, visualization, writing-original draft: D.X.; investigation: X.S.; investigation: A.P.L.; investigation: Y.Z.; writing-review and editing: X.B.; resources: C.-g.Z. investigation: A.M.; investigation: Y.L. conceptualization, supervision, funding acquisition, writing-original draft, project administration, writing-review and editing: X.L. All authors have read and agreed to the published version of the manuscript.

## Declaration of interests

The authors declare no competing interests.

## STAR★Methods

### Key resources table

**Table:** 

REAGENT or RESOURCE	SOURCE	IDENTIFIER
**Antibodies**		

Anti-Histone H3 (di methyl K36) antibody	Abcam	Cat# ab9049; RRID: AB_1280939
Normal Rabbit IgG	Cell Signaling Technology	Cat# 2729; RRID: AB_1031062
DYKDDDDK-Tag(3B9) Mouse mAb	Abmart	Cat# M20008; RRID: AB_2713960
SMYD2 (D14H7) Rabbit Monoclonal Antibody	Cell Signaling Technology	Cat# 9734; RRID: AB_10889559
SARM1 antibody	GeneTex	Cat# GTX131411; RRID: AB_2886480
Anti-Histone H3 antibody	Abcam	Cat# ab1791; RRID: AB_302613
β-Actin Rabbit mAb	ABclonal	Cat# AC026; RRID: AB_2768234
anti-myosin heavy chain (MHC) antibody	DSHB	Cat# MF20; RRID: AB_2147781

**Bacterial strains**		

HT115	Caenorhabditis Genetics Center	DE3
*E. coli* OP50	Caenorhabditis Genetics Center	xu363

**Chemicals, peptides, and recombinant proteins**		

Methylviologen (paraquat)	RHAWN	Cat# R000267
Nicotinamide	Sigma	Cat# 72340

**Critical commercial assays**		

NAD^+^/NADH Assay Kit	Beyotime	Cat# S0175
MitoSOX™	Invitrogen	Cat# M36008

**Experimental models: Cell lines**		

Human HEK293T	Shanghai Institutes for Biological Sciences	CSTR:19375.09.3101HUMSCSP502
mouse C2C12	Shanghai Institutes for Biological Sciences	CSTR:19375.09.3101MOUSCSP505

**Experimental models: Organisms/strains**		

Mouse: C57BL/6J	Chen YL et al.^72^	N/A
*C. elegans*: wild type	Caenorhabditis Genetics Center	N2
*C. elegans*: *set-18(gk334)*	Caenorhabditis Genetics Center	VC767
*C. elegans*: *zcIs14* (P_*myo-3*_::mito::GFP)	Caenorhabditis Genetics Center	SJ4103
*C. elegans*: *zcIs13* (*hsp-6p*::GFP)	Caenorhabditis Genetics Center	SJ4100
*C. elegans*: *tir-1(syb4237)*	sunybiotech	PHX4237
*C. elegans*: *tir-1(syb4226)*	sunybiotech	PHX4226
*C. elegans*: *syb3463* (SET-18::GFP)	sunybiotech	PHX3463
*C. elegans*: *set-18(gk334);tir-1*(*syb4237)*	This paper	XXL0017
*C. elegans*: *set-18(gk334);tir-1(syb4226)*	This paper	XXL0016
*C. elegans*: *P*_*myo3*_*::mito::Grx1-roGFP2*	Qiao X et al.^73^	N/A
*C. elegans*: *set-18(gk334)[P*_*myo3*_*::mito::Grx1-roGFP2]*	This paper	XXL2001
*C. elegans*: *P*_*myo3*_*::cyto::Grx1-roGFP2*	Qiao X et al.^73^	N/A
*C. elegans*: *set-18(gk334)[P*_*myo3*_*::cyto::Grx1-roGFP2]*	This paper	XXL2002
*C. elegans*: *set-18(gk334)[P*_*set-18*_*::SET-18::mCherry;rol-6(su1006)]*	This paper	XXL0035
*C. elegans*: *syb3463*(SET-18::GFP)*[P*_*tir-1d*_*::TIR-1d::mCherry;rol-6(su1006)]*	This paper	XXL0014
*C. elegans*: *tir-1(syb4226)[P*_*tir-1d*_*::TIR-1d::mCherry;rol-6(su1006)]*	This paper	XXL0027
*C. elegans*: *tir-1(syb4226)[P*_*myo-2*_*::TIR-1d::mCherry;rol-6(su1006)]*	This paper	XXL0028
*C. elegans*: *N2[P*_*tir-1d*_*::TIR-1d::mCherry;rol-6(su1006)]*	This paper	XXL0029
*C. elegans*: *set-18(gk334)[P*_*tir-1d*_*::TIR-1d::mCherry;rol-6(su1006)]*	This paper	XXL0030

**Oligonucleotides**		

List of primers and other oligonucleotides, see Table S7	This paper	N/A

**Recombinant DNA**		

P3Xflag-SET-18	This paper	N/A
PGL4.2-*ptir-1a*	This paper	N/A
PGL4.2-*ptir-1b*	This paper	N/A
PGL4.2-*ptir-1c*	This paper	N/A
PGL4.2-*ptir-1d*	This paper	N/A
PGL4.2-*ptir-1e*	This paper	N/A
PGL4.2-*ptir-1d (-491bp ∼ +101bp)*	This paper	N/A
PGL4.2-*ptir-1d (-999bp ∼ +101bp)*	This paper	N/A
PGL4.2-*ptir-1d (-1553bp ∼ +101bp)*	This paper	N/A
PEGFP-N1-SARM1	This paper	N/A

**Software and algorithms**		

Graphpad Prism 8	GraphPad Software, Inc.	https://www.graphpad.com; RRID:SCR_002798
ImageJ	NIH	https://ImageJ.nih.gov/ij/; RRID: SCR_003070

### Experimental model and study participant details

#### *C. elegans* strains

The following *C. elegans* strains were used in this study: N2, wild type; VC767, *set-18(gk334)*; SJ4103, *zcIs14* (*P*_*myo-3*_::mito::GFP); SJ4100, *zcIs13* (*hsp-6p*::GFP) were provided by Caenorhabditis Genetic Center (CGC). PHX4237, *tir-1(syb4237)*; PHX4226, *tir-1(syb4226)*; PHX3463, *syb3463* (SET-18::GFP) were constructed by Suzhou Shangyuan Biotechnology (sunybiotech). XXL0017, s*et-18(gk334);tir-1*(*syb4237)*; XXL0016, s*et-18(gk334);tir-1*(*syb4226)*; XXL2001, *set-18(gk334)[P*_*myo3*_*::mito::Grx1-roGFP2]*; XXL2002, *set-18(gk334)[P*_*myo3*_*::cyto::Grx1-roGFP2]* were generated through standard genetic crosses and confirmed by PCR genotyping. XXL0035, *set-18(gk334)[P*_*set-18*_*::SET-18::mCherry;rol-6(su1006)]*; XXL0014, *syb3463*(SET-18::GFP)*[P*_*tir-1d*_*::TIR-1d::mCherry;rol-6(su1006)]*; XXL0027, *tir-1*(*syb4226)[P*_*tir-1d*_*::TIR-1d::mCherry;rol-6(su1006)]*; XXL0028, *tir-1*(*syb4226)[P*_*myo-2*_*::TIR-1d::mCherry;rol-6(su1006)]*; XXL0029, N2*[P*_*tir-1d*_*::TIR-1d::mCherry;rol-6(su1006)]*; XXL0030, *set-18(gk334)[P*_*tir-1d*_*::TIR-1d::mCherry;rol-6(su1006)]* were generated by standard microinjection and confirmed by PCR genotyping. All strains were grown at 20 °C and maintained according to established general protocols, unless otherwise stated.^74^

#### Mammalian cell lines and aged C2C12 myotubes

Human HEK293T cells (CSTR:19375.09.3101HUMSCSP502) and mouse C2C12 myoblasts (CSTR:19375.09.3101MOUSCSP505) were obtained from Shanghai Institutes for Biological Sciences and cultured as previously described.^75^^,^^76^ Cell lines STR (Short Tandem Repeat) identification has been conducted. Cells were regularly tested for mycoplasma contamination by mycoplasma detection kit. C2C12 myoblasts were differentiated to be myotubes by switching growth medium into differentiation medium (DM medium), which 10% fetal bovine serum was replaced with 2% horse serum (Gibco, USA). The aging (atrophy) of myotubes was induced by treating them with 700 μM H_2_O_2_ (Sigma-Aldrich, Germany) in DM medium for 24 h.

#### Mice

Male C57BL/6J mice were maintained under a standard 12 h alternate light/dark cycle with free access to food and water. The temperature and humidity in the room were maintained at 20 ± 2°C and 50 ± 5%, respectively. Forelimb muscle tissues were collected from mice at 6–8 weeks (young) or 20 months (old) of age following standard housing and maintenance. All mouse experiments were conducted in accordance with the protocols for animal use, treatment, and euthanasia approved by the Animal Care Committee of Northeast Normal University (Authorization number: 202301119).

### Method details

#### *C. elegans* transgenes

The GFP sequence of pPD95.75 plasmid was replaced by mCherry; and then using general cloning techniques, the genome sequence of *set-18* gene with *set-18* promoter, *tir-1d* gene with *tir-1d* promoter or *myo-2* promoter were cloned into pPD95.75 to construct plasmids *P*_*set-18*_*::SET-18::mCherry*, *P*_*tir-1d*_*::TIR-1d::mCherry* and *P*_*myo-2*_*::TIR-1d::mCherry*. All cloned sequences were confirmed by sequencing before use. 20 ng/μL of each of recombinant plasmid was co-injected with 50 ng/μL of marker plasmids pRF4 (*rol-6*) into the germline of young adult hermaphrodite of N2, *set-18*^−/−^, or *tir-1*^*ΔTIR*^, respectively.

#### RNA interference (RNAi) in *C. elegans*

HT115 bacteria expressing dsRNAs that target the specific gene were grown in Lysogeny broth Lennox medium containing 100 μg/mL ampicillin and 12.5 μg/mL tetracycline overnight with shaking at 37 °C. Overnight cultures were seeded onto nematode growth medium (NGM) plates containing 100 μg/mL ampicillin and 4 mM isopropyl 1-thio-β-*d*-galactopyranoside (IPTG) and incubated and dried for 1–2 days at room temperature, after which synchronized L1 larva were transferred to the bacterial lawns and allowed to grow until the stages we needed. All of RNAi sequences are shown in Table S7.

#### Gene knockdown and overexpression in C2C12 myotubes

The small interfering RNA (siRNA) duplex oligoribonucleotides targeting *smyd2* were synthesized by GenePharma (Shanghai, China). The cDNA of *sarm1* was obtained by RT-PCR and ligated into pEGFP-N1 to construct overexpression vector of *sarm1*. Using GP-transfect-Mate Reagent (GenePharma, China), *smyd2* siRNAs were transfected with or without *sarm1* overexpression plasmids into differentiated C2C12 myotubes according to the manual. The non-targeting siRNA and empty pEGFP-N1vector were used as negative controls. All of siRNA sequences are shown in Table S7.

#### Analysis of mitochondrial and cytoplasmic ROS in *C. elegans*

The transgenic worms that respectively express *Pmyo3::mito::Grx1-roGFP2* and *Pmyo3::cyto::Grx1-roGFP2* were loaded into a black-walled 96-well plate and detected by fluorescence microplate reader (Tecan, Switzerland) with 488 nm and 405 nm excitation filter and 535 nm emission filter according to previous publication.^29^^,^^31^ The ratios of fluorescence intensity of oxidized Grx1-roGFP2 (405 nm) to reduced Grx1-roGFP2 (488 nm) represented the ROS level in mitochondria and cytoplasm of worms, respectively. Grx1-roGFP2-expressing lines on the wild-type background are used as controls. Corrections for the intestinal autofluorescence in the transgenic strains were made by subtracting the 535 nm emission autofluorescence of the matched wild-types after 405 nm and 488 nm excitation, respectively, from that of Grx1-roGFP2-expressing lines.

$$\frac{Oxidized}{\text{Reduced}}Grx1-roGFP2=\frac{Grx1-roGFP2\;Ex405Em535-N2\;Ex405Em535}{Grx1-roGFP2\;Ex488Em535-N2\;Ex488Em535}$$

#### Analysis of mitochondrial ROS in C2C12 myotubes

The differentiated C2C12 myotubes cultured on coverslips were incubated with 5 μM mitoSOX Red Mitochondrial Superoxide Indicator (Invitrogen, USA) at 37 °C and 5% CO2. Then, they were fixed with cold 4% paraformaldehyde (PFA) and counterstained the nuclei with DAPI. The stained myotubes were detected and photographed by confocal microscopy (Carl Zeiss LSM880, Germany) with the identical exposure settings. The red fluorescence intensity that determined mitochondrial ROS level were analyzed by ImageJ.

#### NAD^+^ assays

The NAD^+^ level was measured using NAD^+^/NADH Assay Kit (Beyotime, China) according to the manufacturer’s instructions. The collected worms or differentiated C2C12 myotubes were homogenized after adding the extracting solution and finally detected the absorbance of mixed liquid at 450 nm to analysis NAD^+^ level. The values were normalized to the total protein concentration.

#### RT-qPCR

The total RNAs in worms or C2C12 myotubes were extracted using Trizol reagent (Takara, China); and they were utilized to generate cDNAs by Reverse Transcription System (Promega, USA). Using the cDNAs as template, the quantitative PCR (qPCR) was operated on QuantStudio 3 Real-Time PCR System (Thermo, USA) with the SYBR Green Real-time PCR Master Mix (TaKaRa, China). *act-1* and *β-actin* were used as the internal references in worms and C2C12 myotubes, respectively. The primers used for RT-qPCR were listed in Table S7.

#### Chromatin immunoprecipitation (ChIP)-qPCR

According to previous publications,^12^ the worms or C2C12 myotubes were fixed with 1% formaldehyde and lysed and sonicated with Ultrasonic breaker (SCIENTZ-IID, China). H3K36me2 was immunoprecipitated from lysate using rabbit H3K36me2 ChIP-grade (ab9049, Abcam) or rabbit IgG (#2729, Cell Signaling Technology) antibodies and protein A + G Magnetic Beads (16–663, Millipore). Crosslinks were reversed by incubation for 12 h at 65°C. DNA fragments were extracted with phenol-chlorophorm isoamylalcohol and precipitated with ethanol. RT-qPCR experiments were performed as described above. The primers used in ChIP-qPCR were shown in Table S7.

#### *C. elegans* mitochondrial morphology analysis

The worms with expressing *P*_*myo-3*_::mito::GFP were mounted onto agar pads and paralyzed with levamisole (Sigma). The mitochondrial networks of muscle cells were photographed by confocal microscopy (Carl Zeiss LSM880, Germany). According to previous publications,^28^ the mitochondrial morphology of a muscle cell was classified as follows: “tubular”, a majority of long interconnected mitochondrial networks; “intermediate”, a combination of interconnected mitochondrial networks along with some smaller fragmented mitochondria; “fragmented”, a majority of short or sparse small round mitochondria.

#### Analysis of *C. elegans* mitochondrial abundance

The mitochondrial content were measured by analyzing fluorescence intensities of mitochondrial GFP reporter in muscle cells. The worms with expressing *P*_*myo-3*_::mito::GFP were anesthetized with levamisole on agar pads and photographed by confocal microscopy (Carl Zeiss LSM880, Germany). The GFP fluorescence intensities were quantified by ImageJ.

The mtDNA content was relatively quantified by the ratio of mtDNA/nDNA copy numbers in worms. This ratio was determined by qPCR using the primers of mitochondrial gene MTCE.26 and nuclear gene *act-3*, respectively. The primer sequences were shown in Table S7.

#### Analysis of TIR-1d::mCherry and SET-18::GFP expressions

The transgenic worms *tir-1*^*ΔTIR*^[*P*_*tir-1*_::TIR-1d::mCherry+*rol-6(su1006)*] and SET-18::GFP[*P*_*tir-1*_::TIR-1d::mCherry+*rol-6(su1006)*] were used to analysis the TIR-1d expression levels and detect the co-location of TIR-1d::mCherry and SET-18::GFP expressions, respectively. The worms were anesthetized with levamisole on agar pads and photographed by confocal microscopy (Carl Zeiss LSM880, Germany) with the identical exposure settings. The fluorescence intensity of TIR-1d::mCherry was quantified by ImageJ.

#### Western blot

Worms, C2C12 myotubes or muscle tissues of mouse forelimb were lysed in lysis buffer. The proteins of lysate were separated by sodium dodecyl sulfate polyacrylamide gel electrophoresis (SDS-PAGE) and then transferred to PVDF transfer membrane (Merck Millipore), followed by incubation with primary antibodies and horseradish peroxidase-conjugated secondary antibodies. The targeted proteins were visualized using enhanced chemiluminescence ECL reagent (GE Healthcare). Tanon 5500 high-definition low-illumination CCD system (Tanon FLI Capture v.1.02) was used to capture chemiluminescent signals. The primary antibodies used in this study were as follows: anti-Flag (M20008, Abmart), anti-SMYD2 (#9734, Cell Signaling Technology), anti-SARM1 (GTX131411, GeneTex), anti-H3 (ab1791, Abcam) and anti-H3K36me2 (ab9049, Abcam). β-actin (AC026, ABclonal) was used as internal loading control. The blotting intensities were quantified by densitometry analysis.

#### Luciferase reporter assays

The sequences of *tir-1* isoforms’ promoters and the distinct sequences of *tir-1d* promoter were amplified and inserted into the firefly luciferase reporter plasmid pGL4.20, respectively. The cDNA of *set-18* was cloned into p3xFLAG-CMV to construct Flag-SET-18 overexpression vector. Using polyethylenimine (PEI), 200 ng of luciferase reporter plasmid and 500 ng of Flag-SET-18 vector were co-transfected with 50 ng of Renilla luciferase plasmid (internal reference) into human HEK293T cells in 24-well plates. After transfection for 48 h, cell lysates were collected to measure luciferase activity by Dual-Luciferase Reporter Assay System (Promega, China) according to the manufacturer’s instructions. The expression levels of Flag-SET-18 in cell lysates were confirmed by Western blot using Flag antibody.

#### *C. elegans* lifespan and mtROS-defense assays

The *C. elegans* lifespan and mtROS-defense ability were analyzed by performing survival assays of the worms cultured on normal NGM and the medium containing 2 mM paraquate (PQ), respectively. During assays, the fluorodeoxyuridine (FUDR, Sigma) was used to prevent worm reproduction. At least 90 synchronized young adult worms were used per conditions and scored every other day. All survival experiments were performed at 20°C unless stated otherwise. Worms were count as censored in case of internal hatching, crawling off and bursting. If worms did not respond to touches with a platinum pick, they were considered as dead.

#### *C. elegans* pharyngeal pumping rate and body bend frequency

Using stereoscopic microscope, the pharyngeal pumping rate of worms were measured by counting the number of contractions in the terminal bulb of pharynx per 30 s. To determine body bend frequency, individual worms were transferred to M9 buffer and the number of body bends in a 60 s interval was counted. One body bend was defined as complete bending of the worm body in one direction to the outermost angle and back to the initial posture.

#### MHC staining, diameter and fusion index analysis of C2C12 myotubes

The C2C12 myotubes were incubated with anti-myosin heavy chain (MHC) antibody (MF20, DSHB) followed by fluorescent secondary antibody (C2181, Sigma Chemical) to identify MHC expression. The nuclei of myotubes were counterstained with DAPI (D1306, Invitrogen). The stained MHC and nuclei were detected and photographed by confocal microscopy (Carl Zeiss LSM880, Germany). The mean of myotubes diameter were quantified by ImageJ. The fusion index of myotubes was calculated as the value of the number of the nuclei present in myotubes divided by the number of total nuclei in the stained MHC^+^ cells.

### Quantification and statistical analysis

For *C. elegans* lifespan and mtROS-defense assays, *p-values* were determined by *log*-*rank* test. For worm mitochondrial morphology analysis, *p-values* were determined by *chi*-square. For analysis of ROS, NAD^+^ and ATP levels, assays of qPCR, luciferase activity, fluorescence intensities and blotting densitometry, measurement of pharyngeal pumping rate and body bend frequency of worms, and detection of diameter and fusion index of C2C12 myotubes, *p-values* were calculated using Student’s *t* test for comparison between two groups and one-way ANOVA for comparison among multiple groups. Statistical significance was defined for *p* values, ∗, *p* < 0.05, ∗∗, *p* < 0.01, and ∗∗∗, *p* < 0.001.
