# Supplementary material for: Histone methyltransferase SET-18/SMYD2-mediated activation of NADase TIR-1d/SARM1 increases mtROS to promote aging
Source: iScience. 2026 Jan 7;29(2):114649. doi: 10.1016/j.isci.2026.114649 (PMC12857417; doi:10.1016/j.isci.2026.114649)
Supplement: Document S1. Figures S1–S14 and Tables S1–S7 [file mmc1.pdf]

iScience, Volume *29*

Supplemental information

Histone methyltransferase SET-18/SMYD2-mediated activation of NADase TIR-1d/SARM1 increases mtROS to promote aging

Dongxue Xue, Xin Su, Aaron Pambu Lelo, Yongjun Zhang, Xueqing Ba, Cheng-gang Zou, Aohe Ma, Yao Liu, and Xiaoxue Li

Supplemental Information

Histone methyltransferase SET-18/SMYD2-mediated activation of NADase TIR-1d/SARM1 increases mtROS to promote aging

Dongxue Xue, Xin Su, Aaron Pambu Lelo, Yongjun Zhang, Xueqing Ba, Cheng-gang Zou, Aohe Ma, Yao Liu, Xiaoxue Li

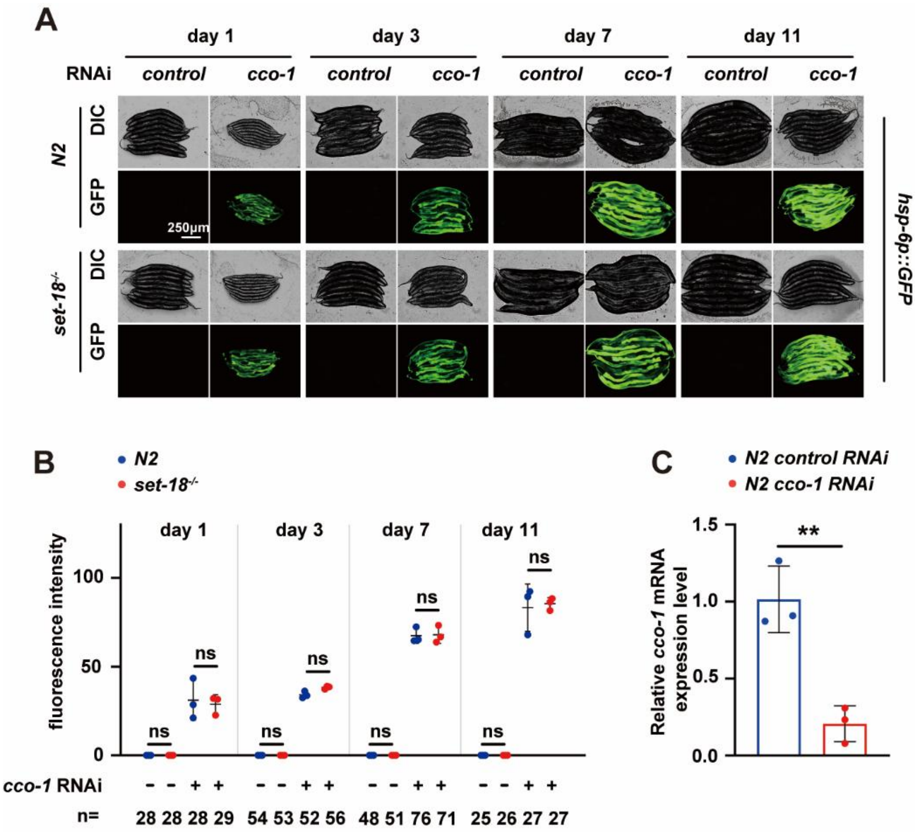

Supplementary Figure S1. UPR^{mt} is not changed by loss of *set-18* through the whole worm life. Related to Figure 1.

N2 worms and *set-18(gk334)* mutants (*set-18*$^{-/-}$) with expressing *hsp-6p*::GFP at young (day 1 and day 3) and old (day 7 and day 11) ages were treated with or without *cco-1* RNAi. *control RNAi*, empty vector. **(A)** The representative images of *hsp-6p*::GFP expression. Scale bar 250 µm. **(B)** The analysis of *hsp-6p*::GFP fluorescence intensities. n, the number of worms used for quantitative analysis. *Cco-1* RNAi: day 1: p (*set-18*$^{-/-}$ vs. N2) = 0.7723, day 3: p (*set-18*$^{-/-}$ vs. N2) = 0.0506, day 7: p (*set-18*$^{-/-}$ vs. N2) = 0.8990, day 11: p (*set-18*$^{-/-}$ vs. N2) = 0.7969; student's *t-test*. **(C)** The efficiency of *cco-1* RNAi in N2 worms. The mRNA level of *cco-1* was measured by RT-qPCR and normalized to that of *control RNAi*. *act-1* was used as an internal reference. Error bars represent SEM. **p (*N2 cco-1 RNAi* vs. *N2 control RNAi*) = 0.0047; student's *t-test*.

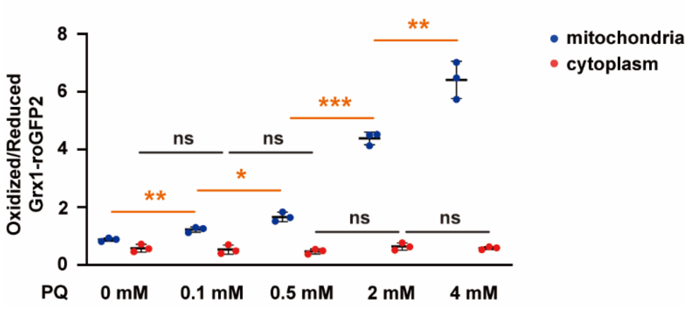

Supplementary Figure S2. The *C. elegans* mtROS accumulation is specifically increased by paraquate (PQ) treatment in dose-dependent manner. Related to Figure 1.

N2 worms with expressing P_{myo3}::mito::Grx1-roGFP2 and P_{myo3}::cyto::Grx1-roGFP2 were treated with 0 mM, 0.1 mM, 0.5 mM, 2 mM and 4 mM paraquate (PQ), respectively. The mitochondrial and cytoplasmic ROS levels were assessed using the ratio of oxidized Grx1-roGFP2 to reduced Grx1-roGFP2. Error bars represent SEM. **mtROS**: **p (0.1 mM vs. 0 mM) = 0.0055, *p (0.5 mM vs. 0.1 mM) = 0.0149, ***p (2 mM vs. 0.5 mM) < 0.0001, **p (4 mM vs. 2 mM) = 0.0067; **cytoROS**: p (0.1 mM vs. 0 mM) = 0.6966, p (0.5 mM vs. 0.1 mM) = 0.5631, p (2 mM vs. 0.5 mM) = 0.1257, p (4 mM vs. 2 mM) = 0.5350; one-way ANOVA.

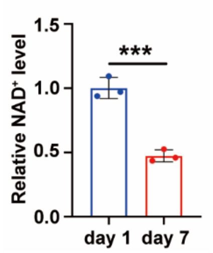

Supplementary Figure S3. The NAD$^+$ level declined in old-aged worms. Related to Figure 2.

The NAD$^+$ level of N2 worms at day 7 was detected by microplate reader and normalized to that of day 1. Error bars represent SEM. n = 3 biological replicates. ***p (day 7 vs. day 1) = 0.0006; student's *t-test*.

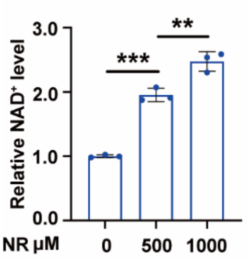

Supplementary Figure S4. NR treatment elevated the NAD$^+$ levels of worms in a dose-dependent manner. Related to Figure 2.

The NAD$^+$ level of N2 worms treated with 500 μM and 1000 μM NR were detected by microplate reader and normalized to that of 0 μM NR. Error bars represent SEM. n = 3 biological replicates. ***p (500 μM vs. 0 μM) = 0.0001, **p (1000 μM vs. 500 μM) = 0.0079; one-way ANOVA.

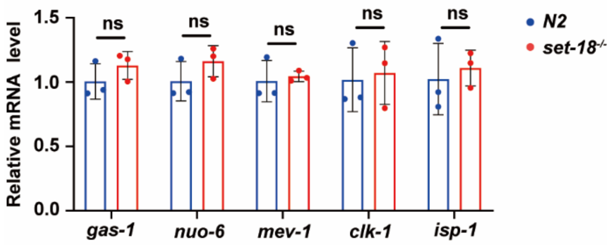

Supplementary Figure S5. The mRNA levels of mitochondrial electron transport chain (ETC) genes were not altered by loss of *set-18*. Related to Figure 2.

The mRNA levels of NADH dehydrogenase *gas-1*, NADH ubiquinone oxidoreductase *nuo-6*, succinate dehydrogenase *mev-1*, ubiquinone *clk-1* and ubiquinol-cytochrome c reductase *isp-1* in N2 and *set-18* mutants were detected by RT-qPCR and normalized to those of N2. *act-1* was used as an internal reference. Error bars represent SEM. ***Gas-1***: p (*set-18*$^{-/-}$ vs. N2) = 0.2874, ***nuo-6***: p (*set-18*$^{-/-}$ vs. N2) = 0.2397, ***mev-1***: p (*set-18*$^{-/-}$ vs. N2) = 0.7200, ***clk-1***: p (*set-18*$^{-/-}$ vs. N2) = 0.8034, ***isp-1***: p (*set-18*$^{-/-}$ vs. N2) = 0.6549; student's *t-test*.

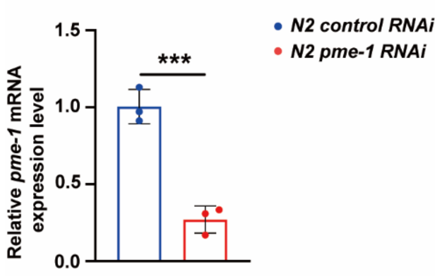

Supplementary Figure S6. The efficiency of *pme-1* RNAi. Related to Figure 2.

N2 worms were treated with *pme-1* RNAi. *control RNAi*, empty vector. The mRNA level of *pme-1* was measured by RT-qPCR and normalized to that of *control RNAi*. *act-1* was used as an internal reference. Error bars represent SEM. ***p (*N2 pme-1 RNAi* vs. *N2 control RNAi*) = 0.0009; student’s *t-test*.

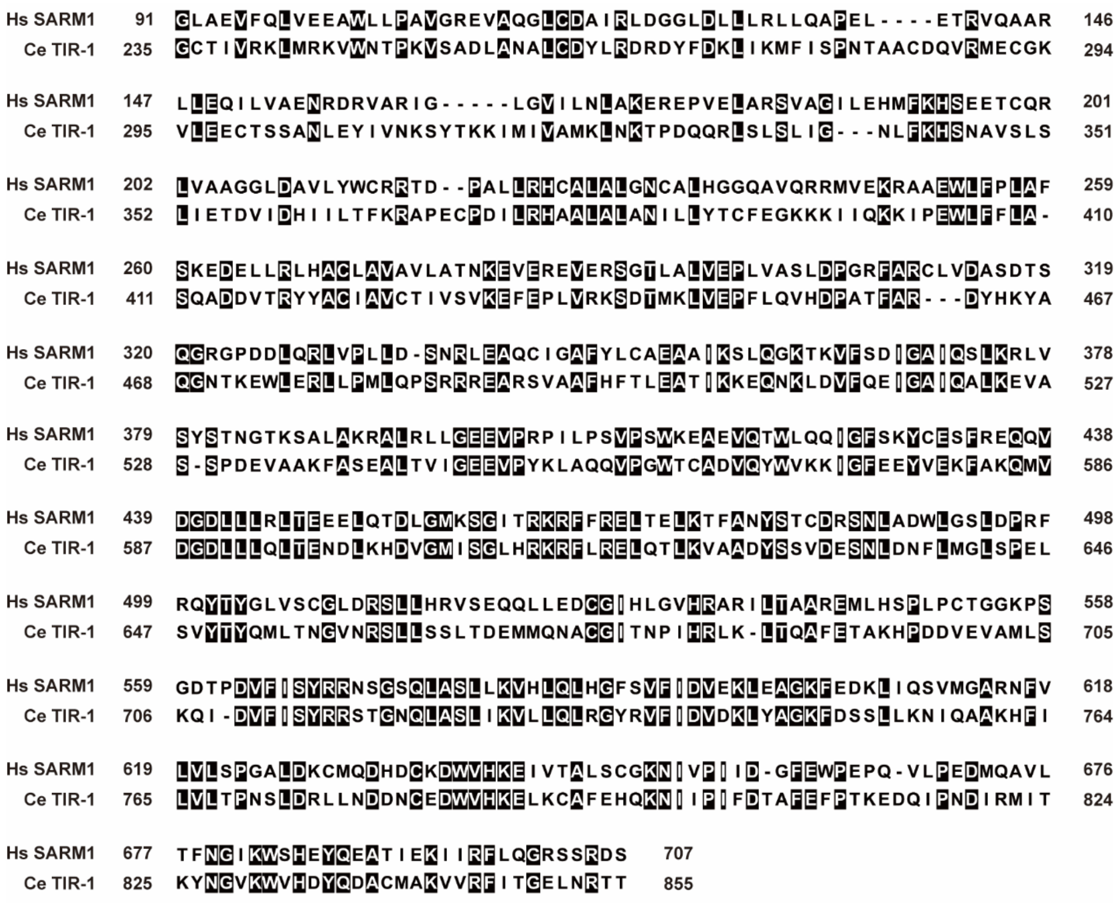

Supplementary Figure S7. The alignment of amino acid sequences between human SARM1 (Hs SARM1) and *C. elegans* TIR-1 (Ce TIR-1). Related to Figure 3.

The identical residues were shaded in black.

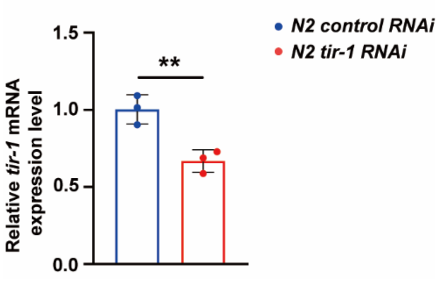

Supplementary Figure S8. The efficiency of *tir-1* RNAi. Related to Figure 3.

N2 worms were treated by *tir-1* RNAi. *control RNAi*, empty vector. The mRNA level of *tir-1* was measured by RT-qPCR and normalized to that of *control RNAi*. *act-1* was used as an internal reference. Error bars represented SEM. **p (*N2 tir-1 RNAi* vs. *N2 control RNAi*) = 0.0084; student's *t-test*.

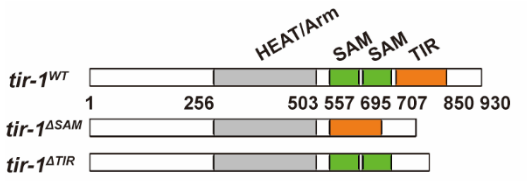

Supplementary Figure S9. The diagram showing that SAM and TIR domain of TIR-1 are deleted in *tir-1*$^{\Delta SAM}$ and *tir-1*$^{\Delta TIR}$ mutants, respectively. Related to Figure 3.

SAM domains (green), TIR domain (yellow) and N-terminal domain (gray).

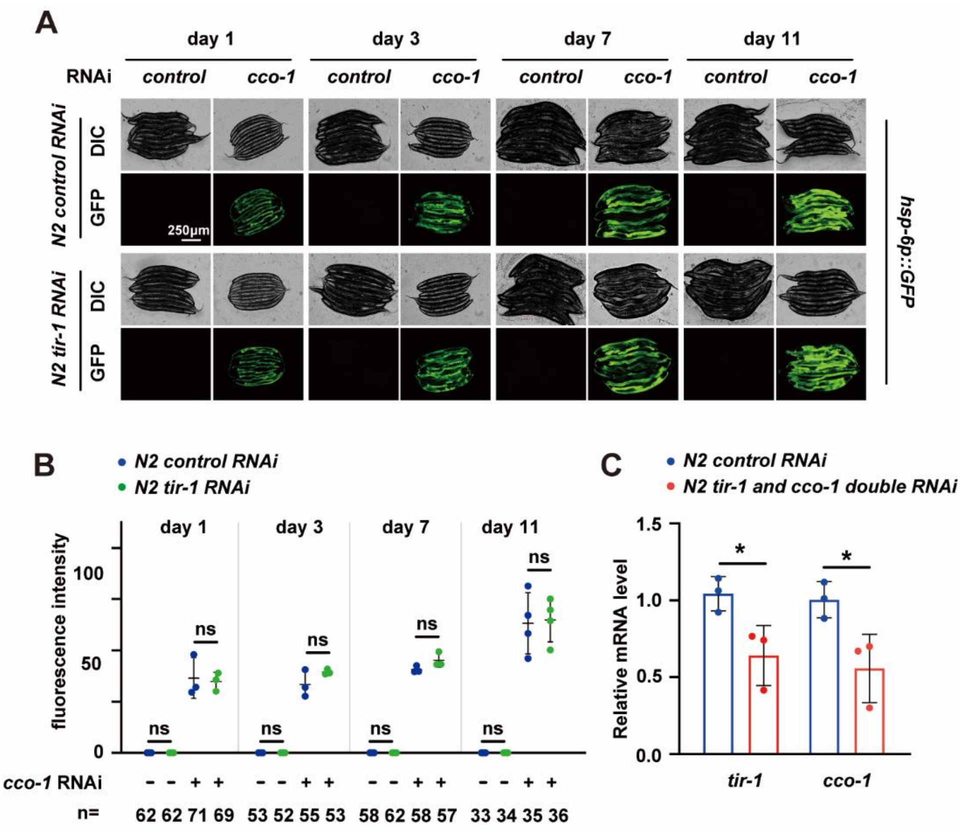

Supplementary Figure S10. UPR^{mt} is not affected by *tir-1* RNAi. Related to Figure 3.

N2 worms with expressing *hsp-6p*::GFP were treated with *tir-1* RNAi and/or *cco-1* RNAi from L1 larva to young (day 1 and day 3) and old (day 7 and day 11) ages, respectively. *control RNAi*, empty vector. **(A)** The representative images of *hsp-6p*::GFP expression. Scale bar 250 μm. **(B)** The analysis of *hsp-6p*::GFP fluorescence intensities. n, the number of worms used for quantitative analysis. *Cco-1* RNAi: day 1: p (*N2 tir-1 RNAi* vs. *N2 control RNAi*) = 0.8021, day 3: p (*N2 tir-1 RNAi* vs. *N2 control RNAi*) = 0.1957, day 7: p (*N2 tir-1 RNAi* vs. *N2 control RNAi*) = 0.1150, day 11: p (*N2 tir-1 RNAi* vs. *N2 control RNAi*) = 0.8643; student's *t-test*. **(C)** The efficiency of *tir-1* and *cco-1* double RNAi in N2 worms. The mRNA levels of *tir-1* and *cco-1* were measured by RT-qPCR and normalized to those of *control RNAi*. *act-1* was used as an internal reference. Error bars represent SEM. ***Tir-1***: *p (*N2 tir-1 and cco-1 double RNAi* vs. *N2 control RNAi*) = 0.0368, ***cco-1***: *p (*N2 tir-1 and cco-1 double RNAi* vs. *N2 control RNAi*) = 0.0369; student's *t-test*.

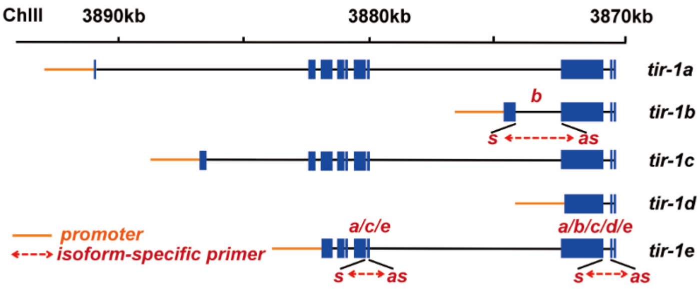

Supplementary Figure S11. The diagram showing the strategies of designing the *tir-1* isoform-specific primers for RT-qPCR and cloning the promoter sequences of each *tir-1* isoforms for luciferase reporter assays. Related to Figure 4.

The genomic position of *tir-1* gene is from 3869590 bp to 3901642 bp on chromosome III. The exons and introns of *tir-1* isoforms (*tir-1a~e*) were represented by boxes and lines, respectively. The sites for designing the isoform-specific (including *tir-1a/c/e*, *tir-1b* and *tir-1a/b/c/d/e*) primers for RT-qPCR were indicated by red dotted lines. s, sense primers; as, antisense primers. The promoter sequences of each *tir-1* isoforms that were cloned into luciferase reporter plasmid displayed with orange solid lines.

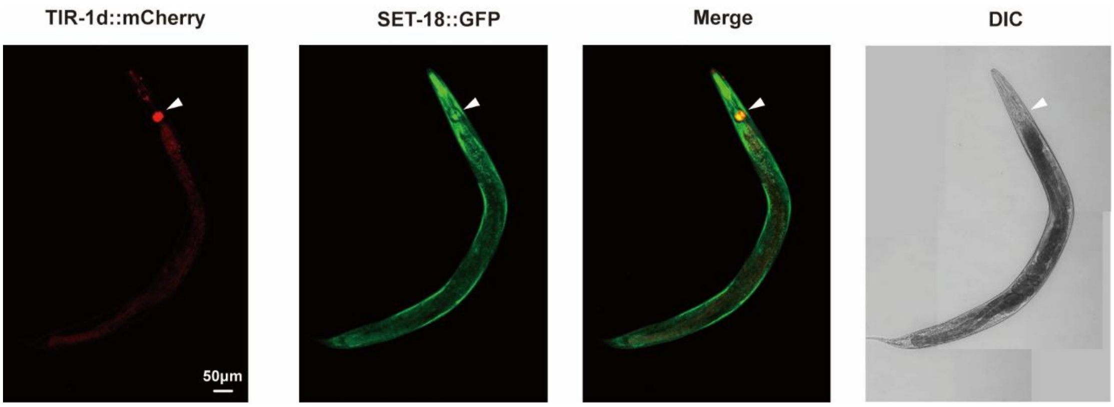

Supplementary Figure S12. The full-size images of worms with expressing TIR-1d::mCherry and SET-18::GFP in Figure 4B. Related to Figure 4.

The expressions of TIR-1d::mCherry and SET-18::GFP were mainly co-localized in worm pharynx (white arrow). Scale bar 50 μm.

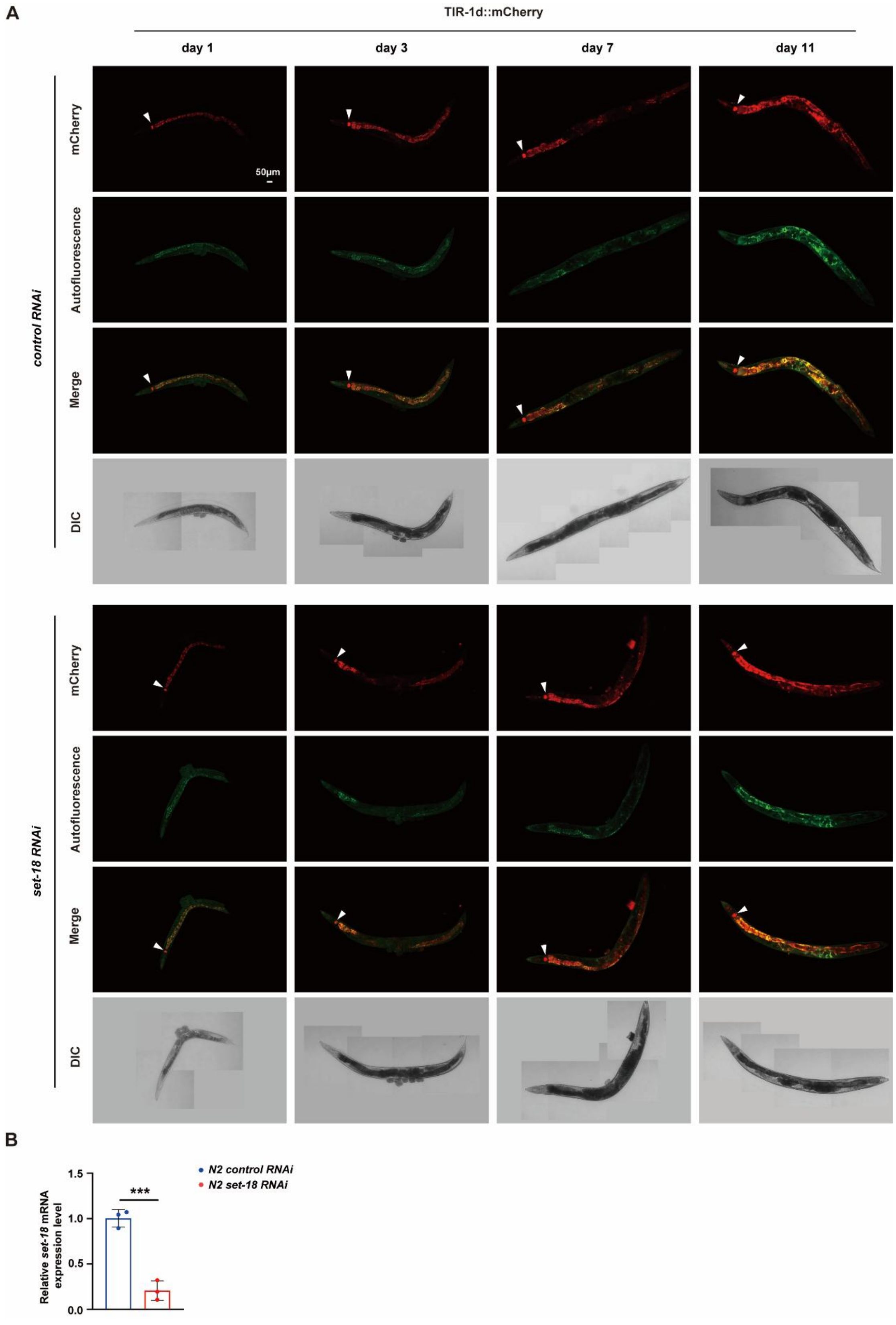

Supplementary Figure S13. The full-size pictures of worms expressing TIR-1d::mCherry with *set-18* RNAi treatment in Figure 4C. Related to Figure 4.

The worms expressing TIR-1d::mCherry were treated with *set-18* RNAi from L1 larva to young

(day1 and day 3) and old (day 7 and day 11) ages, respectively. *control RNAi*, empty vector. **(A)** The representative pictures of whole-body worms taken by confocal microscopy with the identical exposure settings. By using 568 nm in conjunction with 488 nm excitation filters, the intestinal autofluorescence (green) and TIR-1d::mCherry (red) were shown respectively, and the autofluorescence (orange) was discriminated to TIR-1d::mCherry (red) in the merged pictures. Scale bar 50 µm. **(B)** The efficiency of *set-18* RNAi. The mRNA level of *set-18* was measured by RT-qPCR and normalized to that of *control RNAi*. *act-1* was used as an internal reference. Error bars represented SEM. ***p (*N2 set-18 RNAi* vs. *N2 control RNAi*) = 0.0007; student's *t-test*.

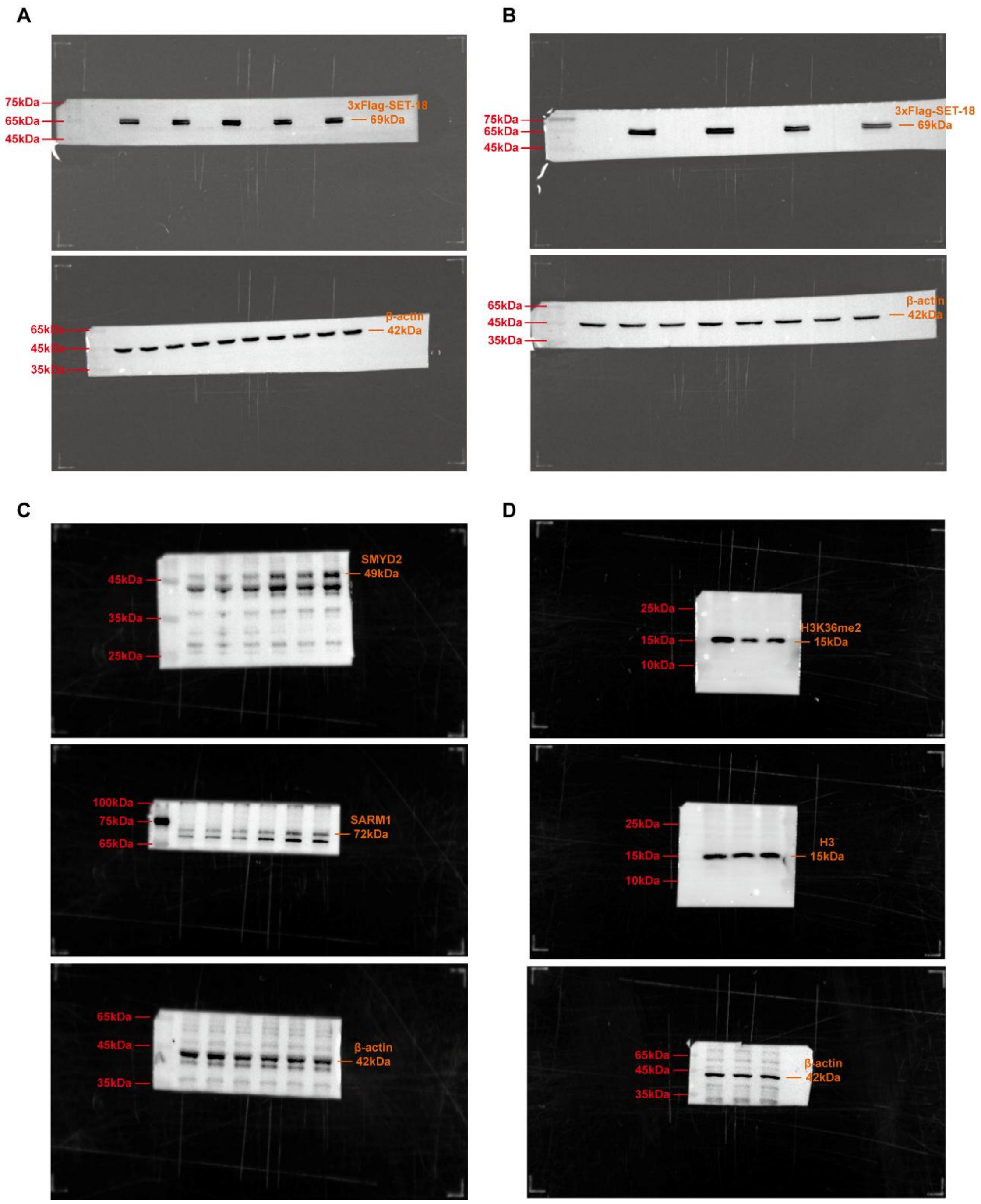

Supplementary Figure S14. Western blot raw data. Related to Figure 4 and Figure 6.

(A-B) The Flag-SET-18 expression level was confirmed by Western blot, using β-actin as an internal reference. Related to Figure 4D-4E. **(C)** The protein levels of SMYD2 and SARM1 in the muscles of C57BL/6J mice were measured by western blots. Related to Figure 6A. **(D)** The global H3K36me2 modification levels in C2C12 myotubes were detected by Western blot. Related to Figure 6D.

Supplementary Table S1. Overexpression of *set-18* abolished the increased viability of *set-18* mutants with paraquate treatment. Related to Figure 1.

Survival assays (2 mM paraquate treatment)	Mean lifespan ± SEM 20 ^{o}C (days)	*P*-value	Total Animals Died/Total
N2	12.898 ± 0.179	-	243/300
set-18$^{-/-}$	16.322 ± 0.031	< 0.0001[a]	251/300
set-18$^{-/-}$ *OE SET-18 1#*	12.814 ± 0.084	< 0.0001[b]	249/300
set-18$^{-/-}$ *OE SET-18 2#*	12.924 ± 0.116	< 0.0001[b]	257/300

"*set-18*$^{-/-}$ *OE SET-18*" *1#* and *2#* were two independent extrachromosomal arrays obtained by co-injecting plasmids $P_{set\text{-}18}$::SET-18::mCherry with *rol-6* marker (pRF4) into *set-18* mutants. All of worms were treated with 2 mM paraquate (PQ), followed by survival assays. The survivals of worms were scored by three independent experiments. The total number of observations equals the number of animals that died plus the number censored. Animals that crawled off the plate, bagged, or burst were censored and were therefore excluded from all analysis. SEM: standard error of the mean. *P*-values were calculated by comparing to as follows: [a]*N2*, [b]*set-18*$^{-/-}$. The *log-rank* test was used for statistical analysis.

Supplementary Table S2. Mutation of *set-18* alleviated the effects of *pme-1* RNAi and NR treatment in enhancing mtROS-defense ability and extending lifespan. Related to Figure 2.

Lifespan assays	Mean lifespan ± SEM 20 °C (days)	*P*-value	Total Animals Died/Total
N2 control RNAi	14.853 ± 0.029	-	261/300
N2 pme-1 RNAi	17.713 ± 0.360	< .0001[a]	254/300
set-18$^{-/-}$ *control RNAi*	17.684 ± 0.391	< .0001[a]	251/300
set-18$^{-/-}$ *pme-1 RNAi*	17.967 ± 0.817	0.6150[b]	249/300
N2	16.051 ± 0.765	-	231/280
N2+NR	18.988 ± 1.179	< .0001[c]	239/280
set-18$^{-/-}$	19.304 ± 0.919	< .0001[c]	240/280
set-18$^{-/-}$*+NR*	19.516 ± 1.368	0.1138[d]	229/280
Survival assays (2 mM paraquate treatment)	Mean lifespan ± SEM 20 °C (days)	*P*-value	Total Animals Died/Total
N2 control RNAi	12.898 ± 0.534	-	265/300
N2 pme-1 RNAi	15.986 ± 0.440	< .0001[a]	283/300
set-18$^{-/-}$ *control RNAi*	15.697 ± 0.448	< .0001[a]	271/300
set-18$^{-/-}$ *pme-1 RNAi*	15.822 ± 0.691	0.5447[b]	273/300
N2	13.413 ± 0.455	-	233/270
N2+NR	15.137 ± 0.664	< .0001[c]	241/270
set-18$^{-/-}$	16.127 ± 0.259	< .0001[c]	234/270
set-18$^{-/-}$*+NR*	15.106 ± 0.384	0.3436[d]	227/270

N2 and *set-18* mutant worms were treated with *pme-1* RNAi and 500 μM NR, respectively. *control RNAi*, empty vector. The survival assays with or without 2 mM paraquat treatment were conducted to analyze worms' mtROS-defense ability and lifespan. The survivals of worms were performed as described in Table S1 and scored by three independent experiments. SEM: standard error of the mean. *P*-values were calculated by comparing to as follows: [a]*N2 control RNAi*, [b]*set-18*$^{-/-}$ *control RNAi*, [c]*N2*, [d]*set-18*$^{-/-}$. The *log-rank* test was used for statistical analysis.

Supplementary Table S3. The extended lifespan and increased mtROS-defense ability of *tir-1*$^{\Delta SAM}$ and *tir-1*$^{\Delta TIR}$ mutants were both abolished by loss of *set-18*. Related to Figure 3.

Lifespan assays	Mean lifespan ± SEM 20 ^{o}C (days)	*P*-value	Total Animals Died/Total
N2	16.005 ± 0.315	-	320/330
tir-1$^{\Delta SAM}$	19.116 ± 0.088	< .0001[a]	311/330
tir-1$^{\Delta TIR}$	19.596 ± 0.137	< .0001[a]	316/330
set-18$^{-/-}$	18.626 ± 0.240	< .0001[a]	305/330
set-18$^{-/-}$;*tir-1*$^{\Delta SAM}$	19.075 ± 0.316	0.7992[b]	311/330
set-18$^{-/-}$;*tir-1*$^{\Delta TIR}$	19.323 ± 0.250	0.0739[b]	311/330
Survival assays (2 mM paraquate treatment)	Mean lifespan ± SEM 20 ^{o}C (days)	*P*-value	Total Animals Died/Total
N2	13.784 ± 0.277	-	261/300
tir-1$^{\Delta SAM}$	17.233 ± 0.187	< .0001[a]	258/300
tir-1$^{\Delta TIR}$	16.883 ± 0.051	< .0001[a]	273/300
set-18$^{-/-}$	16.574 ± 0.255	< .0001[a]	274/300
set-18$^{-/-}$;*tir-1*$^{\Delta SAM}$	17.178 ± 0.198	0.5401[b]	272/300
set-18$^{-/-}$;*tir-1*$^{\Delta TIR}$	17.256 ± 0.220	0.1230[b]	272/300

Using CRISPR-Cas9 technique, the *set-18*$^{-/-}$;*tir-1*$^{\Delta SAM}$ and *set-18*$^{-/-}$;*tir-1*$^{\Delta TIR}$ double mutants were constructed by deleting SAM and TIR domain of TIR-1 in *set-18*$^{-/-}$ mutants, respectively. The survival assays with or without 2 mM paraquat treatment were conducted to analyze worms' mtROS-defense ability and lifespan. The survivals of worms were performed as described in Table S1 and scored by three independent experiments. SEM: standard error of the mean. *P*-values were calculated by comparing to as follows: [a]*N2*, [b]*set-18*$^{-/-}$. The *log-rank* test was used for statistical analysis.

Supplementary Table S4. Rescue of TIR-1d in pharynx attenuated the extended lifespan and enhanced mtROS-defense ability of *tir-1* mutants. Related to Figure 5.

Lifespan assays	Mean lifespan ± SEM 20 °C (days)	*P*-value	Total Animals Died/Total
N2	15.178 ± 0.104	-	236/270
tir-1$^{\Delta TIR}$	18.558 ± 0.086	< .0001[a]	235/270
tir-1$^{\Delta TIR}$*[*$P_{tir\text{-}1d}$*::TIR-1d] 1#*	16.843 ± 0.168	< .0001[b]	229/270
tir-1$^{\Delta TIR}$*[*$P_{tir\text{-}1d}$*::TIR-1d] 2#*	16.898 ± 0.126	< .0001[b]	222/270
tir-1$^{\Delta TIR}$*[*$P_{myo\text{-}2}$*::TIR-1d] 1#*	16.812 ± 0.012	< .0001[b]	229/270
tir-1$^{\Delta TIR}$*[*$P_{myo\text{-}2}$*::TIR-1d] 2#*	17.146 ± 0.158	< .0001[b]	226/270
Survival assays (2 mM paraquate treatment)	Mean lifespan ± SEM 20 °C (days)	*P*-value	Total Animals Died/Total
N2	13.352 ± 0.288	-	246/270
tir-1$^{\Delta TIR}$	16.465 ± 0.143	< .0001[a]	243/270
tir-1$^{\Delta TIR}$*[*$P_{tir\text{-}1d}$*::TIR-1d] 1#*	14.985 ± 0.147	< .0001[b]	251/270
tir-1$^{\Delta TIR}$*[*$P_{tir\text{-}1d}$*::TIR-1d] 2#*	14.840 ± 0.207	< .0001[b]	225/270
tir-1$^{\Delta TIR}$*[*$P_{myo\text{-}2}$*::TIR-1d] 1#*	15.001 ± 0.129	< .0001[b]	240/270
tir-1$^{\Delta TIR}$*[*$P_{myo\text{-}2}$*::TIR-1d] 2#*	14.815 ± 0.074	< .0001[b]	245/270

The plasmids $P_{tir\text{-}1d}$::TIR-1d::mCherry and $P_{myo\text{-}2}$::TIR-1d::mCherry were respectively co-injected with *rol-6* marker (pRF4) into *tir-1*$^{\Delta TIR}$ mutants to rescue TIR-1d expression driven by its own promoter and pharynx-specific *myo-2* promoter. *1#* and *2#* were two independent extrachromosomal arrays. The survival assays with or without 2 mM paraquat treatment were conducted to analyze worms' mtROS-defense ability and lifespan. The survivals of worms were performed as described in Table S1 and scored by three independent experiments. SEM: standard error of the mean. *P*-values were calculated by comparing to as follows: [a]*N2*, [b]*tir-1*$^{\Delta TIR}$. The *log-rank* test was used for statistical analysis.

Supplementary Table S5. The extended lifespan and enhanced mtROS-defense ability of *set-18* mutants are attenuated by overexpression of TIR-1d. Related to Figure 5.

Lifespan assays	Mean lifespan ± SEM 20 °C (days)	*P*-value	Total Animals Died/Total
N2	15.610 ± 0.126	-	312/330
set-18$^{-/-}$	18.221 ± 0.271	< .0001[a]	311/330
N2 OE TIR-1d 1#	13.897 ± 0.137	< .0001[a]	313/330
N2 OE TIR-1d 2#	13.695 ± 0.275	< .0001[a]	305/330
set-18$^{-/-}$ *OE TIR-1d 1#*	16.441 ± 0.270	< .0001[b]	313/330
set-18$^{-/-}$ *OE TIR-1d 2#*	16.268 ± 0.303	< .0001[b]	313/330
Survival assays (2 mM paraquate treatment)	Mean lifespan ± SEM 20 °C (days)	*P*-value	Total Animals Died/Total
N2	12.992 ± 0.257	-	298/330
set-18$^{-/-}$	15.493 ± 0.135	< .0001[a]	313/330
N2 OE TIR-1d 1#	11.595 ± 0.240	< .0001[a]	296/330
N2 OE TIR-1d 2#	11.759 ± 0.161	< .0001[a]	310/330
set-18$^{-/-}$ *OE TIR-1d 1#*	13.908 ± 0.226	< .0001[b]	297/330
set-18$^{-/-}$ *OE TIR-1d 2#*	13.578 ± 0.158	< .0001[b]	300/330

"*N2 OE TIR-1d*" and "*set-18*$^{-/-}$ *OE TIR-1d*" were obtained by co-injecting plasmid $P_{tir\text{-}1d}$::TIR-1d::mCherry with *rol-6* marker (pRF4) to N2 and *set-18* mutant worms, respectively. *1#* and *2#* were two independent extrachromosomal arrays. The survival assays with or without 2 mM paraquat treatment were conducted to analyze worms' mtROS-defense ability and lifespan. The survivals of worms were performed as described in Table S1 and scored by three independent experiments. SEM: standard error of the mean. *P*-values were calculated by comparing to as follows: [a]*N2*, [b]*set-18*$^{-/-}$. All statistical analysis was carried out using Graphpad Prism 8 software. The *log-rank* test was used for statistical analysis.

Supplementary Table S6. The enhanced muscle functions of *set-18* mutants at old age (day 11) was abolished by overexpression of TIR-1d. Related to Figure 5.

Body bending frequency	Mean ± SEM (bending numbers /1 min)	*P*-value
N2	75.733 ± 1.707	-
set-18$^{-/-}$	86.133 ± 2.537	0.0005[a]
N2 OE TIR-1d 1#	69.200 ± 1.617	0.0109[a]
N2 OE TIR-1d 2#	69.800 ± 2.616	0.0276[a]
set-18$^{-/-}$ *OE TIR-1d 1#*	76.033 ± 4.278	0.0042[b]
set-18$^{-/-}$ *OE TIR-1d 2#*	76.333 ± 4.284	0.0043[b]
**Pharyngeal pump rate**	**Mean ± SEM (contraction numbers /30 sec)**	***P*-value**
N2	37.133 ± 1.648	-
set-18$^{-/-}$	43.367 ± 1.328	0.0003[a]
N2 OE TIR-1d 1#	32.600 ± 0.709	0.0107[a]
N2 OE TIR-1d 2#	31.033 ± 1.097	0.0006[a]
set-18$^{-/-}$ *OE TIR-1d 1#*	38.700 ± 1.701	0.0226[b]
set-18$^{-/-}$ *OE TIR-1d 2#*	38.367 ± 1.670	0.0031[b]

The strains "*N2 OE TIR-1d*" and "*set-18*$^{-/-}$ *OE TIR-1d*" *1#* and *2#* were identical to the ones used in Table S5. 30 of worms at old age (day 11) were randomly selected from each strain to detect their muscle functions. The frequency of body bend was represented by average numbers of worm body bends during one minute. The rate of pharyngeal pumping was assessed by calculating the number of worm pharyngeal contractions during 30 seconds. SEM: standard error of the mean. *P*-values were calculated by comparing to as follows: [a]*N2*, [b]*set-18*$^{-/-}$. The one-way ANOVA was used for statistical analysis.

Supplementary Table S7. The primers used for RT-qPCR, RNAi and ChIP-qPCRs.

Primer Names	Sequences (5'-3')
Primers for RT-qPCR in *C. elegans*	
act-1 Forward	TCGGTATGGGACAGAAGGAC
act-1 Reverse	CATCCCAGTTGGTGACGATA
tir-1a/c/e Forward	GAAAGACTGCTCCCAATG
tir-1a/c/e Reverse	TCTGCTCCTTCTTAATAGTTG
tir-1b Forward	CCACCCGTATGCAAAGGA
tir-1b Reverse	TCGCCGATAACCGTGAGT
tir-1 Forward	GCTCGAAAGACTGCTCCC
tir-1 Reverse	GCTTGGATTGCACCGATT
tir-1a/b/c/d/e Forward	GGGTTCACGACTATCAGG
tir-1a/b/c/d/e Reverse	AGAAGGCATTTCTTTGGT
pme-1 Forward	GTTTTCATAAAATTAAGGAG
pme-1 Reverse	TCAACTGGATCAATACCC
qns-1 Forward	TTGACGAGATTGAAGAAA
qns-1 Reverse	TGAATAGCAGGAGTTGAG
pnc-1 Forward	TGGAGGCAATACCCGAACA
pnc-1 Reverse	CGATTCCCAAGCCAAGAT
nmat-1 Forward	ATGGAGGATGCCAAGTAC
nmat-1 Reverse	TTCGCATAACCATCACTG
nmat-2 Forward	AGGTCGCCAGGTCACATC
nmat-2 Reverse	AAGCGTTGGTTTATTGTTGT
sir-2.1 Forward	CTTGGCAAAATAATGACG
sir-2.1 Reverse	TTGTAATGGAGTGGCACC
myo-3 Forward	CCAGAAGATGGATTCGTCGCC
myo-3 Reverse	TGGCATTGATCTTTTTTGACGG
unc-54 Forward	GGTTTTGGAGGATCAATCTAAGCC
unc-54 Reverse	CCCTTGGTGGCGGTGATTT
unc-15 Forward	TCGCTCAACGCAAGGTCG
unc-15 Reverse	ACCTCGGCTTGCTTACGGG
lev-11 Forward	ACTTGAAGTCACTTGAGGTGTCCG
lev-11 Reverse	ACGGGTCTCAGCCTCCTTCAGT
pat-10 Forward	CTTTTCGACAAGGAGGGTAACG
pat-10 Reverse	CGTCCTCGTCAATCTCGTCG

mup-2 Forward	GGCTCAGGCTAAGGGAGGAAA
mup-2 Reverse	AGACGGCGGCCAAGAAGTT
act-3 Forward	TGCGACATTGATATCCGTAAGG
act-3 Reverse	GGTGGTTCTCCGGAAAGAA
MTCE.26 Forward	GGTTGTGGGACTAGGTGAACA
MTCE.26 Reverse	CAGGGTGCCCCATTGTTCTT
gas-1 Forward	CAATTTAAGACCGGAGGT
gas-1 Reverse	AACTGGATCGGAATAGGC
nuo-6 Forward	GAGACTCACGGAAAGACC
nuo-6 Reverse	AAATCTCCTTGACACGAT
mev-1 Forward	TCAATAATGTTGGACAGAT
mev-1 Reverse	CTGGCAAGAGTTGAAGAC
clk-1 Forward	CTCGGTGTCGGTTCAGCA
clk-1 Reverse	GGATCGTCGGCAAGGAGT
isp-1 Forward	TTTTGCAGTCAAAGTGATC
isp-1 Reverse	CGGAAAGCAGCGACATTA
Primers for RT-qPCR in Mouse	
β-actin Forward	CTAAGGCCAACCGTGAAAAG
β-actin Reverse	ACCAGAGGCATACAGGGACA
smyd2 Forward	TCGCCAGGAAAGAAGGAT
smyd2 Reverse	AAACAACCATGGAGGAGC
sarm1 Forward	GCTGTTGCTCGATTCGTC
sarm1 Reverse	CACCTTGGTCTTTCCCTGT
MHC Forward	CACCTCCACAGCACAGACAG
MHC Reverse	ACCTTGGCCATGTGATTGTT
Atrogin-1 Forward	CAGCTTCGTGAGCGACCTC
Atrogin-1 Reverse	GGCAGTCGAGAAGTCCAGTC
Murf-1 Forward	GTGTGAGGTGCCTACTTGCTC
Murf-1 Reverse	GCTCAGTCTTCTGTCCTTGGA
Primers for RNAi (dsRNA) in *C. elegans*	
cco-1 Forward	ATTGTTCGCATTTTCAAGGG
cco-1 Reverse	ATAGTGGAACACGAGACCCG
pme-1 Forward	ACTAGAATCGATCGTGAAAGCAG
pme-1 Reverse	GTCTATACGTTCAAAATCGTCCG
tir-1 Forward	TTGAAAGACATTGGGAGTTTGAT

tir-1 Reverse	TTCTTGATGGGATTAAGTCCAGA
set-18 Forward	GGGGTACCTAGCAGGAACGTTGGGCTAC
set-18 Reverse	GCTCTAGACGGTCGATGCAATTCTTTTT
	Primers for RNAi (siRNA) in Mouse
NT Forward	CCACCUCUGAUCGAUUUAUdTdT
NT Reverse	dTdTAUAAAUCGAUCAGAGGUGG
SMYD2 1# Forward	CACCAGUUCUACUCCAAGUTT
SMYD2 1# Reverse	ACUUGGAGUAGAACUGGUGTT
SMYD2 2# Forward	GCUACAUCGACCUGCUAUATT
SMYD2 2# Reverse	UAUAGCAGGUCGAUGUAGCTT
Primers for ChIP-qPCR in *C. elegans*	
tir-1d promoter (-41/+101) F	AGATTGGTTTTGAGGAGTACGTTGA
tir-1d promoter (-41/+101) R	AGCTCTCTTAAGAATCGTTTTCGAT
tir-1d promoter (-191/-42) F	AAGCATTAAAAGAAGTGGCCTCCTC
tir-1d promoter (-191/-42) R	TCTTCACCCAATATTGAACATCTGC
tir-1d promoter (-341/-192) F	CTATGACAGTTTCCACTCCTGAACG
tir-1d promoter (-341/-192) R	GGATTGCACCGATTTCCTGAAAATT
tir-1d promoter (-367/-342) F	CGCAACTGTAATAATGAGGTCAACC
tir-1d promoter (-367/-342) R	CCAGAGGAATATGAGATAAGTAATG
tir-1d promoter (-2000/-1850) F	CGTAACCTGCTTCCAAGATTCGCTG
tir-1d promoter (-2000/-1850) R	TGAGAGACTTGAACAGTTTGAATTG
Primers for ChIP-qPCR in Mouse	
sarm1 promoter (-80/+32) F	TCTTTCTCCTCCCAAGGCC
sarm1 promoter (-80/+32) R	TTGTAGGCGGAGAAGAGCA
sarm1 promoter (-114/-220) F	GCGTCTGGTCCATGCTTCTCT
sarm1 promoter (-114/-220) R	CGGTAAAGGTGCTGGGCTA
sarm1 promoter (-260/-361) F	TAGAGGCGGGGCGTCAAA
sarm1 promoter (-260/-361) R	GAACAAAGCGCTGCTCCAAT
sarm1 promoter (-384/-484) F	CTTTACAGCCCTTTCTACAGCATCT
sarm1 promoter (-384/-484) R	AAGCCCTACCCCAGGTCTCCA
sarm1 3'UTR R	GACATCCAAGACAGGGGAAGAACAT
sarm1 3'UTR F	TCCAGGCGTTGTATTGACCCTTATG
